# Cluster Randomized Controlled Trial: Clinical and Cost-Effectiveness of a System of Longer-Term Stroke Care

**DOI:** 10.1161/STROKEAHA.115.008585

**Published:** 2015-07-27

**Authors:** Anne Forster, John Young, Katie Chapman, Jane Nixon, Anita Patel, Ivana Holloway, Kirste Mellish, Shamaila Anwar, Rachel Breen, Martin Knapp, Jenni Murray, Amanda Farrin

**Affiliations:** From the Academic Unit of Elderly Care and Rehabilitation, Bradford Teaching Hospitals NHS Foundation Trust (A. Forster, J.Y., K.C., K.M., R.B.), Leeds Institute of Clinical Trials Research, Clinical Trials Research Unit (J.N., I.H., S.A., A. Farrin), and Leeds Institute of Health Sciences (J.M.), University of Leeds, Leeds, United Kingdom; Institute of Psychiatry, King’s College London, London, United Kingdom (A.P.); and Personal Social Services Research Unit, London School of Economics and Political Science, London, United Kingdom (M.K.).

**Keywords:** cluster randomized controlled trial, community health services, cost-benefit analysis, quality-adjusted life years, rehabilitation, stroke

## Abstract

**Background and Purpose—:**

We developed a new postdischarge system of care comprising a structured assessment covering longer-term problems experienced by patients with stroke and their carers, linked to evidence-based treatment algorithms and reference guides (the longer-term stroke care system of care) to address the poor longer-term recovery experienced by many patients with stroke.

**Methods—:**

A pragmatic, multicentre, cluster randomized controlled trial of this system of care. Eligible patients referred to community-based Stroke Care Coordinators were randomized to receive the new system of care or usual practice. The primary outcome was improved patient psychological well-being (General Health Questionnaire-12) at 6 months; secondary outcomes included functional outcomes for patients, carer outcomes, and cost-effectiveness. Follow-up was through self-completed postal questionnaires at 6 and 12 months.

**Results—:**

Thirty-two stroke services were randomized (29 participated); 800 patients (399 control; 401 intervention) and 208 carers (100 control; 108 intervention) were recruited. In intention to treat analysis, the adjusted difference in patient General Health Questionnaire-12 mean scores at 6 months was −0.6 points (95% confidence interval, −1.8 to 0.7; *P*=0.394) indicating no evidence of statistically significant difference between the groups. Costs of Stroke Care Coordinator inputs, total health and social care costs, and quality-adjusted life year gains at 6 months, 12 months, and over the year were similar between the groups.

**Conclusions—:**

This robust trial demonstrated no benefit in clinical or cost-effectiveness outcomes associated with the new system of care compared with usual Stroke Care Coordinator practice.

**Clinical Trial Registration—:**

URL: http://www.controlled-trials.com. Unique identifier: ISRCTN 67932305.

## Abstract 2

Supplemental Digital Content is available in the text.

The high prevalence and diversity of longer-term problems experienced by patients with stroke and their carers has long been recognized,^1^ but they remain poorly addressed by existing services.^2^ Postdischarge contact with therapy services is associated with improved outcomes.^3,4^ However, these interventions are generally time limited and have little effect on psychosocial outcome. National guidelines^5^ acknowledge that stroke should be regarded as a long-term condition, and the role of a Stroke Care Coordinator (SCC) to facilitate inputs for community-based patients with stroke and their families after initial (usually hospital-based) treatment is a recommended policy.^6,7^ However, procedures and processes of this role are unevenly developed, and there has been no robust evaluation.

Using the Medical Research Council framework for the development and evaluation of complex interventions,^8^ incorporating systematic reviews, qualitative exploration, and intervention modeling, we developed an evidence-based system of care (longer-term stroke [LoTS] care) that aimed to meet the longer-term needs of patients with stroke and their carers living at home. The system of care incorporates a structured assessment focused on patient- and carer-centered problems and is linked to evidenced-based treatment algorithms and a goal and action planner based on a problem-solving approach. We report findings from a trial evaluation of the LoTS care system of care.

## Methods

### Study Design and Participants

The trial was a pragmatic cluster randomized controlled trial of the clinical and cost-effectiveness of the LoTS care system of care delivered by community-based SCCs compared with SCC usual practice. Trial procedures were informed by a survey of SCCs.^9^ The methods have been reported in detail elsewhere^10^ and undertaken after appropriate ethical approval.

A SCC was eligible if the following criteria were fulfilled: a registered healthcare professional with documented experience in stroke care; undertaking a community-based coordinating role for patients with stroke; in contact with patients and coordinating a range of care inputs on behalf of the patient or carer (eg, signposting, carrying out assessments); receiving referrals from an acute stroke service that included a stroke unit fulfilling the Royal College of Physicians audit definition.^11^ A SCC was classified as working in a team if they participated in community multidisciplinary team meetings. Eligibility of the SCC service was confirmed before randomization by completion of a questionnaire and semistructured interview describing the service and client group (these were repeated midway through recruitment and after 12-month follow-up). This information was also used for stratification to provide context for trial implementation and monitor any potential contamination or confounding between the 2 arms of the trial.

In keeping with the pragmatic trial design, patient eligibility criteria were broad. Patients were eligible if they had a confirmed primary diagnosis of a new stroke, were referred to an SCC on discharge home or within 6 weeks of stroke (if not admitted to hospital), and were still waiting for their first community SCC assessment. Patients were excluded if they had a planned permanent admission to, or were already resident in, a nursing or residential care home; or their main requirement was palliative care. Carers were eligible if a participating patient identified them as the main informal carer who provided practical support at least once per week. Written informed consent for baseline and follow-up assessments was obtained from patients (and carers if appropriate) before baseline assessment. In the event that a patient lacked capacity to consent, the patient’s family member, carer, or friend was asked to act as the consultee and provide consultee declaration.

### Randomization and Masking

The unit of randomization was the SCC service randomized on a 1:1 basis to either the control or the intervention group. Randomization was stratified by the quality of the local stroke unit (National Sentinel Stroke Audit median score of 65 based on 2006 data)^11^; annual number of referrals to the SCC service; SCCs working alone or within a community team; and by geographical area. A method of obtaining a balanced randomization from these covariates based on a method by Carter and Hood^12^ was used.

### Control

Patients in services allocated to the control group received the SCC service in accord with the existing local policies and practices. After randomization, the control group SCCs were asked to complete time logs for all patients documenting the number and duration of contacts, and the time spent coordinating actions, note writing, and discussing the patient in multidisciplinary team meetings if these took place.

### Intervention

The SCC services allocated to the intervention group provided care according to the LoTS care system of care. This comprises a framework of 16 structured assessment questions (linked to evidence-based treatment algorithms and reference guides) that directly relate to longer-term stroke problems previously identified by patients with stroke and their carers^13,14^ and related prompts provided in a care plan. The care plan also includes a goal and action planner to be completed after each contact (patients and carers). This system of care was supported by a training program and detailed manual, underpinned by a problem-solving approach (Table 1; details are provided in the online-only Data Supplement). The number of contacts was not specified but determined by the SCCs’ usual practice and patient need. The key principles being that all assessment questions were asked and that goals and action plans were identified and reviewed with service responses being provided as appropriate within the context of the services available and the patient’s own environment.^15^

**Table 1.**
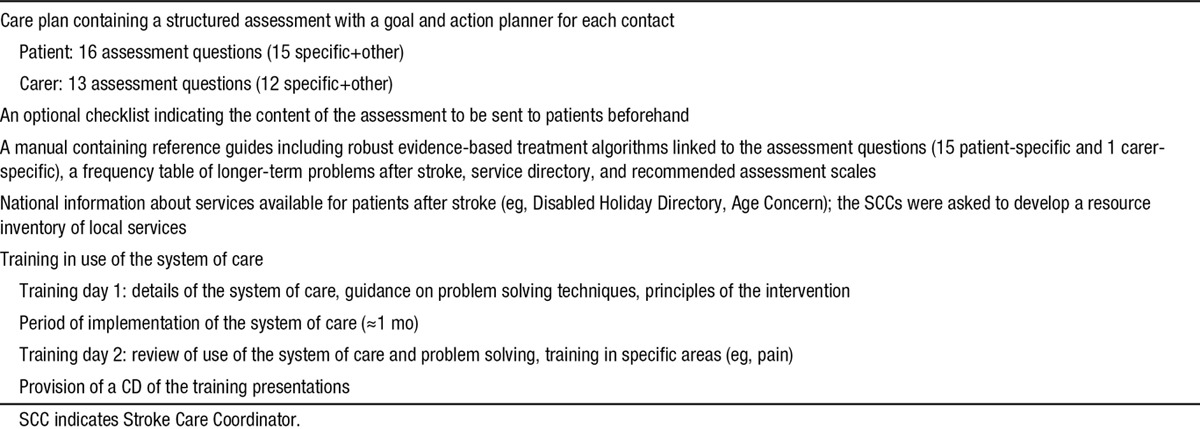
Components of the Longer-Term Stroke Care System of Care

Recruitment was opened 4 to 6 months after the initial training meeting, providing sufficient time for the implementation of the system of care into standard practice. Compliance with the intervention was predefined by the trial team as including at least 12 (75%) of the 16 assessment areas recorded as discussed on the care plan during the first patient contact.

### Procedures

The trial used existing SCC referral pathways as determined during site set-up. The majority of patients were referred to an SCC service through a predischarge inpatient referral. Recruitment of trial participants was by independent research staff blinded as to whether they were recruiting within a control or an intervention service, and the SCCs were unaware which of their patients had consented to participate. This trial design reduced the potential for selection bias from differential recruitment^16^ inherent if the SCCs were responsible for patient identification and minimized likelihood of altering of SCCs’ clinical activity for trial participants.

The research staff collected anonymous screening data (demographic data and modified Rankin Scale score^17^) for all patients referred to a participating SCC. For recruited patients, researchers collected baseline demographic data, including assessment of cognition (6-Item Cognitive Impairment Test),^18^ language ability, prestroke disability (Barthel index^19^), and the 6 items that allow calculation of the Edinburgh stroke case mix adjuster.^20^

Consistent with a patient-centered model of stroke recovery in which adjustment to activity restrictions and participation are regarded as the critical issues, the primary outcome was an assessment of psychological well-being measured by patient reported General Health Questionnaire-12 (GHQ-12)^21^ at 6 months after recruitment, with a secondary outcome at 12 months (for the GHQ-12 higher scores equate to poorer outcomes). This outcome measure is short and easy to complete, consistent with the high prevalence of psychological symptoms after stroke,^13^ and that psychological problems become more prevalent with time.^22^ Other patient reported secondary outcome measures were Frenchay Activities Index (extended activities of daily living),^23^ Barthel Index (activities of daily living),^19^ and European Quality of Life-5 Dimensions (health state).^24,25^ Patients also completed the Longer-term Unmet Needs after Stroke questionnaire (unmet stroke related needs).^26^ Carer-reported outcomes included GHQ-12 and Carer Burden Scale (assessment of carer burden).^27^ A Client Service Receipt Inventory (CSRI^28,29^; use of services prestroke and postdischarge) was completed to inform the economic evaluation. The research staff recorded deaths, emergency outpatient treatment, and hospital admissions at 6 and 12 months post recruitment.

Patient- and carer-reported outcomes were assessed in baseline questionnaires and via postal questionnaires at 6 and 12 months post recruitment. This was supported by postal and telephone reminders if questionnaires were not returned within 2 weeks. If necessary (after postal and telephone reminders), patients were contacted by telephone to complete the primary outcome measure (GHQ-12).

Process data were collected for trial patients after the end of 12 month follow-up so as not to unblind the SCC to trial patients. Time logs were collected for patients in control services. In intervention services, data on the use of the structured assessment and goal and action planner (including time taken) were collected by researchers transcribing the appropriate information from the care plans at site.

### Sample Size

The sample size calculations, based on the primary outcome measure GHQ-12 at 6 months, indicated that recruitment of 800 patients from 40 services would provide 90% power at 5% significance level to detect a clinically relevant difference of 2.5 GHQ-12 points (SD, 7), as reported in a previous study.^30^ This sample size accounted for an estimated 25% loss to follow-up and clustering: the inflation factor of 1.95 was derived from a maximum cluster size of 20 and an intracluster correlation coefficient of no >0.05.^31^ Losses to follow-up were anticipated to increase over time, and interpretation and credibility of results are difficult if losses exceed 30%; therefore, follow-up was limited to 12 months post recruitment and the primary outcome was defined as 6 months.

We were able to identify 32 SCC services which were eligible, willing to participate and provide a principal investigator, and these were randomized. We planned that each service would recruit 25 patients for 18 months, to provide power of 88%, assuming equal cluster size and no >25% loss to follow-up. To minimize unequal recruitment to clusters, the maximum number of patients per service was capped at 45.

### Statistical Methods

Statistical analyses were conducted on the intention to treat population for both primary and secondary analyses. The intention to treat population was defined as all patients registered for active follow-up regardless of noncompliance with the intervention. A per-protocol analysis was also undertaken where major protocol violators and patients not receiving care from SCC were excluded from the analysis. All statistical testing was performed at a 2-sided 5% significance level. Analyses were completed using SAS software version 9.2 (SAS Institute Inc, Cary, NC).

Primary and secondary outcomes were compared between the control and intervention groups using a 2-level multilevel model, with patients nested within stroke services. The models were adjusted for the patient-level covariates (level 1): baseline Barthel Index (prestroke and poststroke), sex, age, living circumstances (living alone versus with carer), stroke severity reflected by speech and language impairment (normal/impaired), baseline 6-Item Cognitive Impairment Test score (normal/impaired cognitive function), and patient baseline score for the outcome measure; and the following stroke unit-level covariates (level 2): quality of stroke unit (National Sentinel Stroke Audit score), referral rate, and SCCs working alone versus within a community multidisciplinary team.

Sensitivity analyses were undertaken to test the robustness of analysis assumptions, including patients who died by assuming worst possible GHQ-12 outcome; only including patients returning postal questionnaires at 6 months (excluding patients who provided primary outcome via telephone call); repeating the analysis without proxy responses; using data collected at 12 months for patients who did not return questionnaires at 6 months, and assuming data missing at random using multiple imputation.

Details of patient deaths and hospital readmissions, unmet needs, carer deaths, any serious adverse events, and related and unexpected serious adverse events were reported for each treatment group. The relationships between adjusted primary outcome and completion of time logs in control or compliance with care plans in intervention were explored graphically.

### Economic Evaluation

The prospective economic evaluation was from both health/social care and societal perspectives linking costs (including SCC inputs) with the GHQ-12 and quality-adjusted life years (QALY) derived from European Quality of Life-5 Dimensions. The primary end point was 6 months, but we also examined findings at 12 months and over the year (full details are provided in the online-only Data Supplement). Unit costs (£, 2010/11 prices) were attached to individual-level resource use quantities (measured retrospectively by self-report) to calculate total costs per participant. Costs are shown in English pounds sterling (£) and can be converted to US dollars using the rate £1=$1.43, based on 2011 purchasing power parities, which equalize the purchasing power of the currencies.^32^ Discounting was unnecessary for the study time frame. We compared costs and QALY gains using multilevel models with baseline covariates, calculating incremental cost-effectiveness ratios only where 1 intervention showed higher costs and better outcomes. We examined the intervention’s probability of cost-effectiveness by constructing cost-effectiveness acceptability curves (5000 bootstrap replications) for threshold ranges of £0 to £2000 for GHQ-12 point gains and £0 to £50 000 for QALY gains.

## Results

The CONSORT diagram is shown in Figure. Thirty-two stroke service clusters were randomized (75% of these involved were team-based rather than individual SCCs); 29 clusters recruited participants (14 control and 15 intervention clusters). Of the 3 services unable to participate, 1 service changed its referral process after randomization; in 1 service, the SCC was absent because of long-term sickness; and in 1 service, we were unable to identify a researcher to undertake recruitment.

**Figure.**
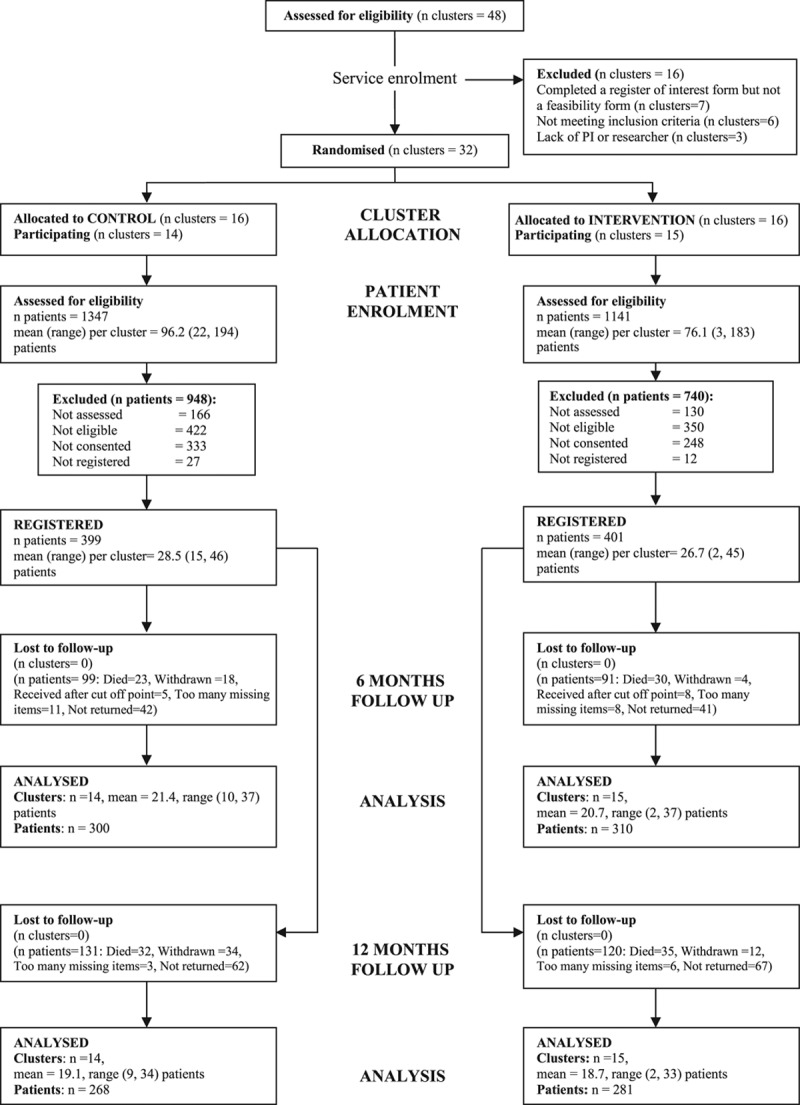
Cluster allocation and patient flow by treatment arm. PI indicates principal investigator.

Between July 1, 2009 and March 31, 2011, 2488 patients were screened; 800 patients (56.3% [800/1420] of eligible patients) and 208 carers were registered into the trial (399 patients/100 carers in the control group; 401 patients/108 carers in the intervention group). Of these, 66 of 800 (8.2%) were by consultee declaration. The study was completed after receipt of the 12-month assessments of the final patients in May 2012. The baseline characteristics of the patients and carers were balanced across the control and intervention groups other than there were more patients with higher education and fewer with language or cognitive impairments in the control group and a difference in length of inpatient stay which was shorter in the control arm. Language and cognitive impairment were accounted for in the statistical modeling. Patient baseline characteristics are shown in Table 2, and carer baseline characteristics are shown in the online-only Data Supplement.

**Table 2.**
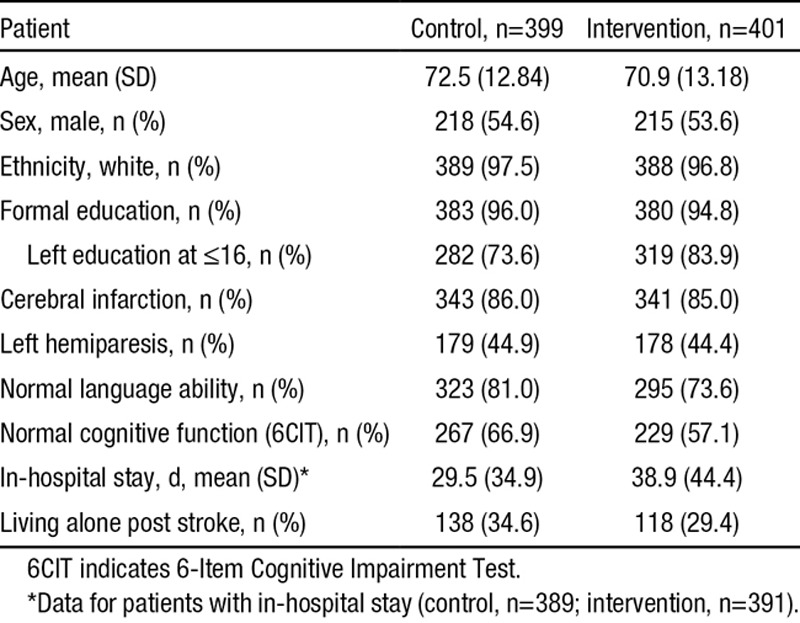
Patient Baseline Demographic and Clinical Details

Of patients registered to the trial, 314 of 399 (78.7%) patients in the control group and 318 of 401 (79.3%) patients in the intervention group received the SCC service. Reasons for not receiving the service included SCC not receiving a referral; patient declining; patient death or patient not contactable. Response rates for patient reported outcomes at 6 months were 75.2% (300/399) in control and 77.3% (310/401) in intervention and at 12 months, 67.2% (268/399) in the control and 70.1% (281/401) in the intervention group. Response rates for carer reported outcomes are provided in the online-only Data Supplement.

There was no evidence of a statistically significant difference for the primary end point. The adjusted GHQ-12 mean score at 6 months was 14.9 (SE, 0.6) points for the control group and 15.5 (SE, 0.6) points for the intervention group, with a difference of −0.6 points (95% confidence interval, −1.8 to 0.7), *P* value of 0.394, and adjusted intracluster correlation coefficients of 0.013 in the control group and 0.025 in the intervention group. Analyses of secondary patient end points also indicated no evidence of statistically significant differences in the Barthel Index, European Quality of Life-5 Dimensions, Frenchay Activities Index at 6 and 12 months, or the GHQ-12 at 12 months (Table 3; unadjusted patient questionnaire scores are provided in the online-only Data Supplement). The number and types of unmet needs reported (Longer-term Unmet Needs after Stroke questionnaire), deaths, hospital readmissions, institutionalization, and treatment on an emergency outpatient basis were similar for both groups (details are provided in the online-only Data Supplement). Between-group comparisons of the adjusted scores for the carer reported GHQ-12 and Carer Burden Scale at 6 and 12 months indicated no evidence of statistical differences for these outcomes (Table 3).

**Table 3.**
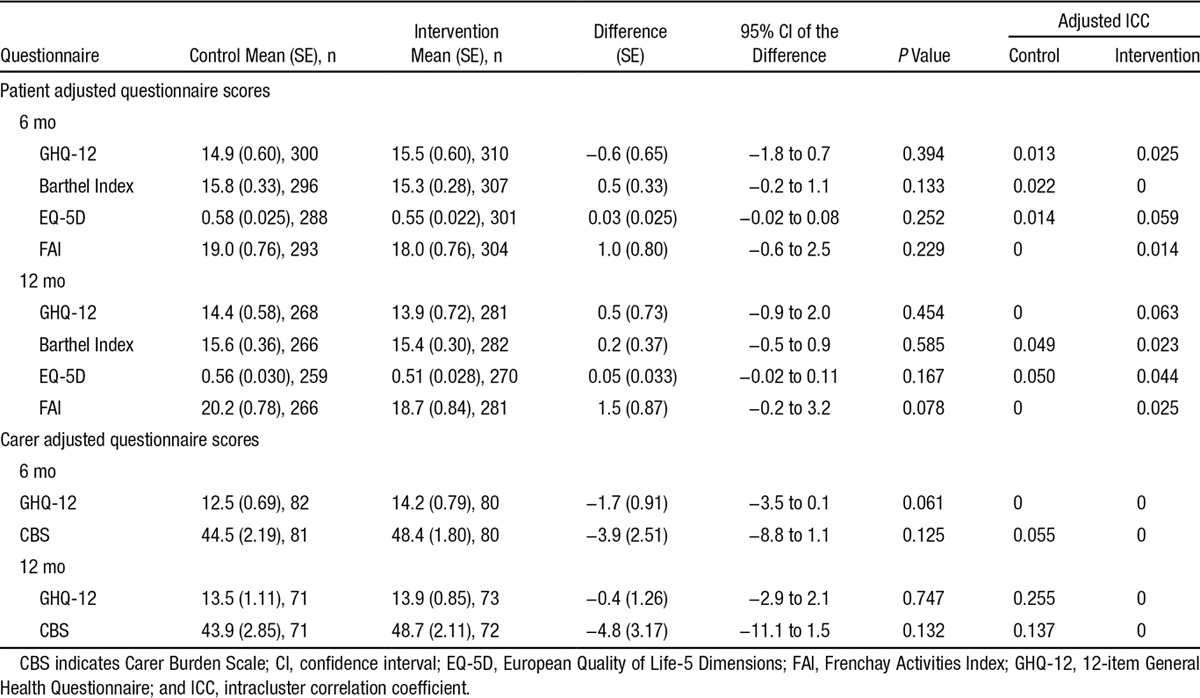
Primary and Secondary Outcomes: Patient- and Carer-Adjusted Questionnaire Scores at 6 and 12 mo

Results of per-protocol analyses (conducted for all patient and carer end points) and sensitivity analyses (conducted on the primary end point) were consistent with results of the intention to treat analyses with no evidence of statistical differences between treatment groups (Table 4).

**Table 4.**
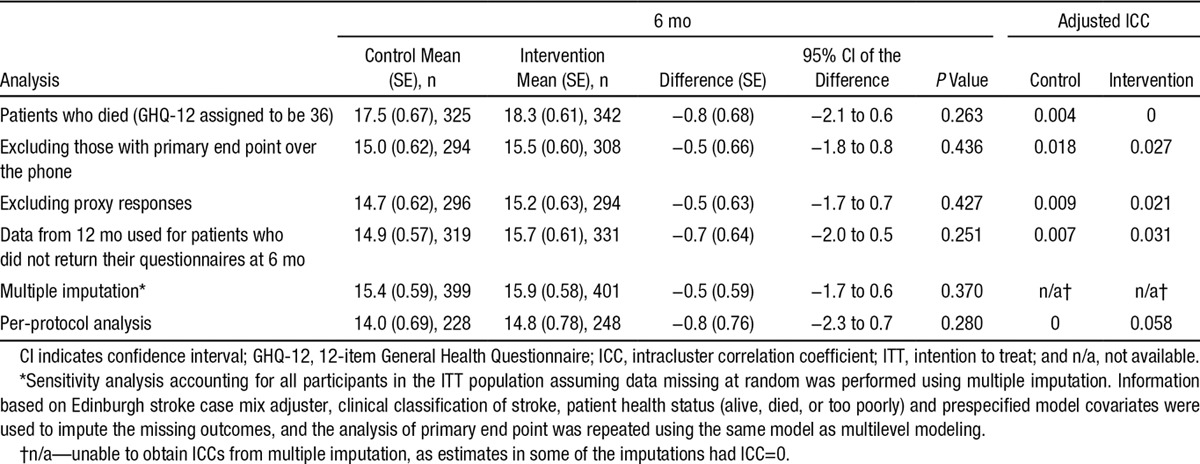
Sensitivity and Per-Protocol Analyses for Primary End Point—Patient-Adjusted GHQ-12 Scores at 6 mo

SCCs completed care plans within the trial period for 280 of 401 (69.8%) patients in the intervention group and time logs for 207 of 399 (51.9%) patients in the control group. In the intervention group, there were 269 of 280 (96.1%) care plans that met the definition of compliance with on average 15 of 16 (93.8%) of assessment areas asked at first contact. From the care plan and time log records, the median number of SCC patient assessment contacts was 2 (range, 1–6) contacts in the intervention group and 2 (range 1–7) contacts in the control group. No linear trend was observed between percentage of compliant care plans or percentage of completed time logs and adjusted mean primary outcome for intervention and control services respectively (Figures and further information presented in the online-only Data Supplement). Review of the semistructured interviews indicated that no control SCCs used a similar structured assessment tool or had access to an evidenced-based treatment manual.

For the economic evaluation, 564 (70.5%) participants had the combination of complete cost and European Quality of Life-5 Dimensions data and 589 (73.6%) had complete cost and GHQ-12 data. Costs of SCC inputs were similar in both groups (mean difference, £42; 95% confidence interval, −30 to 116; Table 5; mean includes zero costs where SCC inputs were not received). There were no differences in mean total health and social care costs (Table 5). Informal care costs increased after baseline and were significantly higher in the intervention group at 6 months, 12 months, and over the year. Although informal care costs fell between the 6- and 12-month assessments in the control group, they increased over the same period in the intervention group. This is reflected in higher total societal costs in the intervention group at 6 and 12 months and over the year (mean difference at 6 months, £1163; 95% confidence interval, 56–3271; Table 5). QALY gains were similar (Table 5). Imputing missing health and social care costs and QALYs at 6 months did not alter conclusions about cost or QALY differences. Incremental cost-effectiveness ratios were unnecessary because no cost–outcome combination suggested statistically significant between-group increases in both costs and outcomes. At 6 months, the intervention had low probabilities of cost effectiveness from both perspectives and for both outcomes, remaining <0.3 for the threshold ranges examined. More details on the economic evaluation are given in the online-only Data Supplement.

**Table 5.**
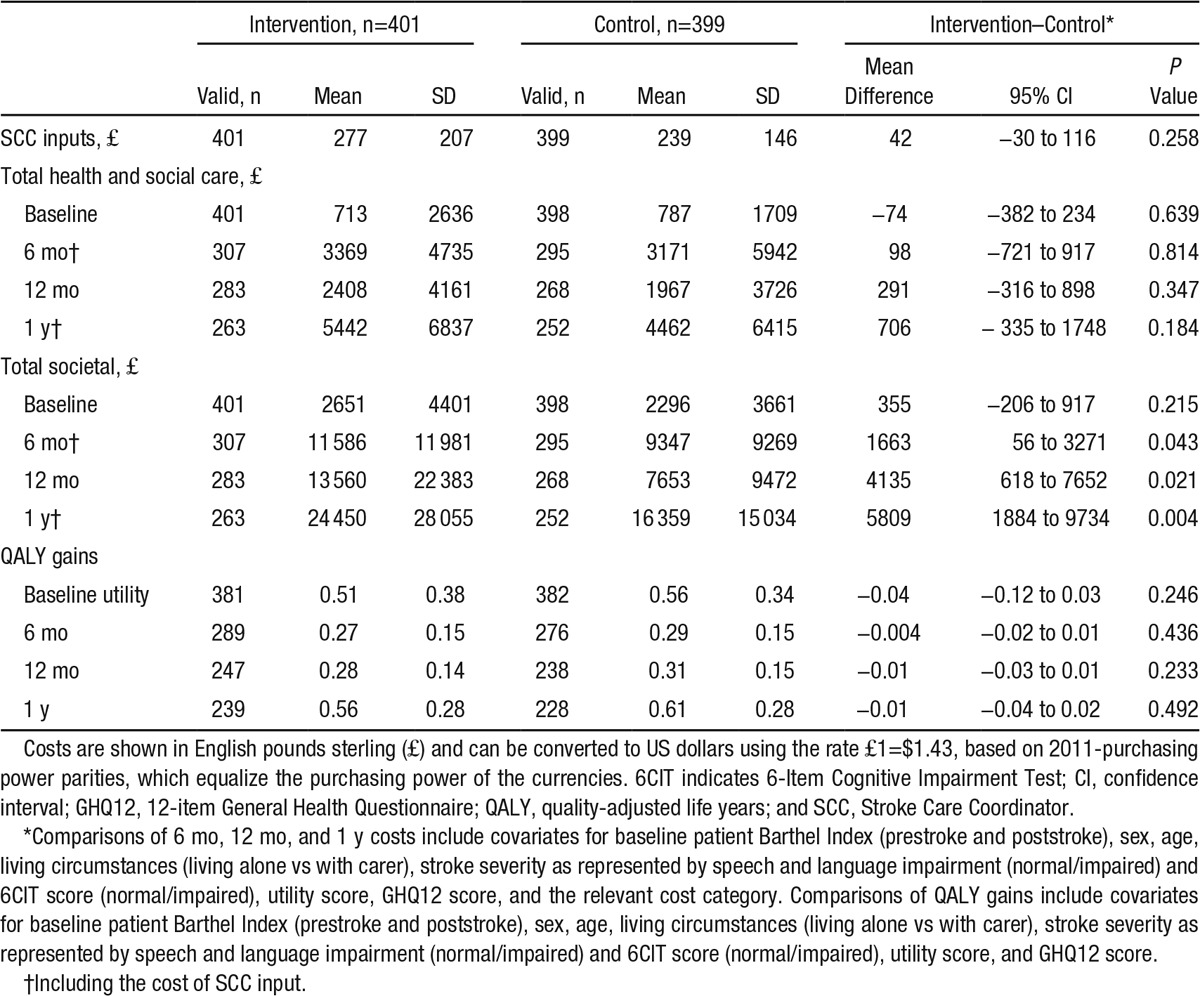
Mean Total Costs at Baseline (for the Previous 3 mo), 6 mo (Previous 6 mo), 12 mo (Previous 6 mo), and Over the Year (£, 2010/11 Prices); QALY Gains at 6 mo (Since Baseline), 12 mo (Since 6 mo), and Over the Year

## Discussion

The LoTS care trial was a pragmatic, multicenter, cluster randomized, controlled trial of a complex intervention designed to provide a structured but individualized care planning process that encompassed the range of problems commonly encountered by people recovering from a stroke and delivered by SCCs working in the community. The main finding was no evidence of statistically significant differences between the control and intervention groups in primary or secondary outcomes in the intention to treat and per-protocol analyses, or in the primary outcome in sensitivity analyses, and no evidence of cost effectiveness from health and social care and societal perspectives.

### Strengths of the Study

The trial followed closely the Medical Research Council guidance on the evaluation of a complex intervention. The trial recruited 800 patients in 29 services across the United Kingdom. This makes it one of the largest stroke rehabilitation trials completed to date. The range of disparate geographical regions ensured a good representation of different healthcare settings optimizing generalizability. The eligibility criteria were kept to a minimum, in keeping with the pragmatic trial design, to ensure that a stroke patient population representative of referrals to SCCs was recruited, including patients with language and cognitive impairment. Of the patients eligible for the trial, 56% were recruited. Comprehensive screening and recruitment data were collected from all participating services, and no evidence for bias in selection procedures was found. The 75% follow-up rate for patients at 6 months as required for the power calculation was achieved. Postal assessment was used to maintain unbiased assessment, problematic in cluster trials where unblinding of a researcher to 1 participant would inevitably result in unblinding of the whole cluster. The intervention documentation (care plan) was so designed that it replaced the intervention SCCs’ previous patient documentation and thus became embedded in their standard practice. A range of sensitivity analyses were undertaken to test robustness of trial results.

### Limitations of the Study

The GHQ12 was selected as an appropriate primary outcome measure that was person-centered and reflected the desirable outcome of well being after stroke rather than the more narrow focus of functional recovery. It is possible that the GHQ12 was insufficiently sensitive to change to identify outcome differences in our study population, and an alternative primary outcome addressing quality of life may have been more appropriate. However, we used a broad range of secondary outcomes all of which demonstrated no differences between treatment arms.

In designing the trial we were concerned about losses to follow-up, which are likely to increase over time and impair the interpretation and credibility of results. We therefore limited follow-up to 12 months post recruitment and defined the primary outcome at 6 months. However, it may be that the intervention had a beneficial effect for a longer period of time, which our assessment schedule would not detect.

### Interpretation

Unlike other chronic diseases in which evidenced-based guidelines for long-term care management are available (eg, heart failure^33^), there has been little research on comprehensive management systems to optimize outcomes for patients with stroke. The need for this is attested by observational studies that describe longer-term stroke outcomes, for example, in a survey of >750 stroke survivors in the United Kingdom 1 to 5 years after the stroke onset,^34^ half reported some unmet needs. This deficiency in care was identified in the specific recommendation of the National Stroke Strategy (England)^7^ and other national guidelines^35,36^ for regular poststroke reviews. The LoTS care system of care was designed to address this recommendation.

Although the structured assessment was delivered to the majority of participants who received the intervention SCC service, it is more difficult to assess the extent to which a change of practice occurred in relation to the problem-solving and goal-setting approaches. The number of patient assessment contacts was comparable with the control services, with a median of 2 contacts in both. This reflects the pretrial survey and existing SSC service models but may be insufficient contact time to meaningfully review goals and develop a problem-solving approach. Although all of the reference guides are evidence-based, the evidence points to more effective interventions for certain problems than for others, thereby potentially weakening the overall effectiveness of this complex intervention. In addition, some needs may only be successfully addressed if appropriate care and treatment are locally available for the problems identified. This may be particularly relevant in relation to psychological problems.^37^ This in turn impinges on our main outcome measure that assessed psychological wellbeing.

### Future Research

There remains a need to develop, implement, and evaluate complex interventions capable of addressing the longer-term needs of people recovering from stroke. Regular review of these needs is an essential component of a longer-term stroke service not only to improve patient care but also to highlight gaps in service provision, which require addressing. The heterogeneity of stroke survivors, who have a wide range of problems and unmet needs, indicates that targeted more bespoke interventions may be the way forward.

## Acknowledgments

We are grateful to all the patients, carers, and stroke services who participated in this trial. Details of participating services are provided in the online-only Data Supplement. The longer-term stroke (LoTS) care trial was a huge collaborative effort, immensely supported by the Stroke Research Networks in the United Kingdom. Our appreciation and gratitude goes to all who supported the development of the intervention, delivery of the training, and implementation of the trial. We thank Peter Wanklyn, Tony Rudd, and Allan House who assisted with the intervention training. We also thank the Consumer Research Advisory Group and the many colleagues in the Academic Unit of Elderly Care and Rehabilitation, Bradford Teaching Hospitals National Health Service Foundation Trust and University of Leeds, and the Clinical Trial Research Unit, University of Leeds and Kings College London, including R. Romeo, M. Heslin, A. Carter, A. Fergusson, and N. Hearfield who provided help and support in the delivery of this trial.

## Sources of Funding

This report presents independent research commissioned by the National Institute for Health Research (NIHR) under its Programme Grants for Applied Research Programme (grant reference number, RP-PG-0606-1128). The views and opinions expressed by the authors in this publication are those of the authors and do not necessarily reflect those of the National Health Service, the NIHR, or the Department of Health. We also acknowledge the funding support of The Stroke Association (TSA 2006/15).

## Disclosures

None.

## Supplementary Material
