# Supplementary material for: Cluster Randomized Controlled Trial: Clinical and Cost-Effectiveness of a System of Longer-Term Stroke Care
Source: Stroke. 2015 Jul 27;46(8):2212–9. doi: 10.1161/STROKEAHA.115.008585 (PMC4512748; doi:10.1161/STROKEAHA.115.008585)

SUPPLEMENTAL MATERIAL

Cluster randomised controlled trial: the clinical and cost-effectiveness of a system of longer-term stroke care

Authored by the LoTS care stroke system of care trial team, who are:

Anne Forster PhD[1], John Young MSc[1], Katie Chapman PhD[1], Jane Nixon PhD[2], Anita Patel PhD[3] Ivana Holloway MSc[2], Kirste Mellish PhD[1], Shamaila Anwar PhD[2], Rachel Breen PhD[1], Martin Knapp PhD[5], Jenni Murray PhD[4], Amanda Farrin MSc[2]

[1] Academic Unit of Elderly Care and Rehabilitation, Bradford Teaching Hospitals NHS Foundation Trust and University of Leeds, UK
[2] Leeds Institute of Clinical Trials Research, Clinical Trials Research Unit, University of Leeds, UK
[3] Institute of Psychiatry, King's College London, UK
[4] Leeds Institute of Health Sciences, University of Leeds, UK
[5] Personal Social Services Research Unit, London School of Economics and Political Science, UK

Correspondence to: Professor Anne Forster, Academic Unit of Elderly Care and Rehabilitation, Temple Bank House, Bradford Royal Infirmary, Bradford, United Kingdom, BD9 6RJ. Email: a.forster@leeds.ac.uk. Tel: +44 1274 383406

Cover title: LoTS care stroke system of care trial

LoTS care stroke system of care trial

Contributions

The major contributors provided the following contributions to this paper: AFo, the conception and design of the study, acquisition and interpretation of data and drafting of this paper and had final responsibility for the decision to submit for publication. JY, the conception and design of the study, interpretation of data and commenting on the draft of this paper. AP, the design of the study, analysis and interpretation of data and drafting of this paper. JN, the conception and design of the study, interpretation of data and commenting on the draft of this paper. MK, the design of the study and commenting on the draft of this paper. IH, analysis and interpretation of data and drafting of this paper. RB, the design of the study and commenting on the draft of this paper. JM, the conception and design of the study and commenting on the draft of this paper. KM, acquisition and interpretation of data and drafting of this paper, KC, acquisition and interpretation of data and commenting on a draft of this paper, SA, data acquisition and commenting on a draft of this paper. AFa, the conception and design of the study, statistical guarantor and acquisition, analysis and interpretation of data and drafting of this paper.

LoTS care Trial Collaboration

Trial Steering Committee. H Rodgers, P Langhorne, A Drummond, J Mant, J Firth, K Hood

This project was funded by a National Institute for Health Research (NIHR) Programme Grant (Grant Reference Number RP-PG-0606-1128). The report of the full programme of research (including this trial) is published in the NIHR Journals Library Volume 2 issue 6. For further information see: http://www.journalslibrary.nihr.ac.uk/pgfar/volume-2/issue-6#abstract December 2014.

The applicants of this grant were: Anne Forster, John Young, Bipin Bhakta, Terry Brady, Jenni Murray, Allan House, Jenny Hewison, Anita Patel, Martin Knapp, Amanda Farrin.

Participating SCCs

Alison Allerton, Susan Altham, Cate Benfold, Michelle Black, Claire Butterworth, Sarah Butterworth, Liz Brown, Yvonne Brown, Bronagh Byrne, Steve Carpenter, Carmen Cartmell, Michael Chivhunga, Mary Downing, Jo Featherstone, Frank Foreman, Debbie Gibbons, Sheila Grimes, Sandra Griffiths, Heather Hazzard, Jenny Hewitson, Grace Hogan, Barbara Holley, Jules Jeffreys, Sandra Leech, Kirstie McCue, Lisa Mewton, Vicki Moore, Nicola Reynolds, Carole Saunders, Susan Targett, Hilary Thompson, Anita Tunstall, Raj Vaithilingam, Susan Wilson, Linda Woollatt

Participating hospitals and PCTs *(number of patients recruited)* and research teams

Airedale General Hospital (20), Paula Sharratt; *Ashford Hospital (16),* Emma Young; *Blackpool Victoria Hospital (27),* J McIlmoyle, Hannah Goddard, Joanne Howard, Carol Jeffs; *Cardiff Royal Infirmary (27),* Catherine Watkins; *Chesterfield Royal Hospital (33),* S Punnoose, J Cooke, Maggie Ball; *Chorley and South Ribble Hospital (12),* Julie Brown, Alison O'Keeffe, Angela Alty, Jackie Purcell, Stephen Duberley; *Christchurch Hospital (6),* Anna Orpen; *Craigavon Hospital (36),* Jane Greene, Diane Crooks, Mary McParland; *Crediton Hospital (1)*, Leigh Barron, Nicola Wedge; *Daisy Hill Hospital (27),* Jane Greene, Mary McParland; *Darent Valley Hospital (27),* P Aghoram, S Hussein, Tracey Daniel, Emily Jay; *Derbyshire County PCT (2)*, Maggie Ball; *Derriford Hospital (20),* Ben Hyams, Nicola Persad, Claire Brown; *Erne Hospital (20),* Celia Diver-Hall; *Fairfield Hospital (3),* Nessa Thomas; *Gloucestershire Royal Hospital (13),* Fiona Davis, Pauline Brown; *Inverclyde Royal*

Hospital (2), Marlene McKinney, Donald Fraser; *Ipswich Hospital (18)*, A Dunthorne, Nicola Rands; *Lymington New Forest Hospital (17),* G Durward, Vanessa Pressly; *Manchester Royal Infirmary (1),* Stephen Duberley; *Mount Gould Hospital (6),* Ben Hyams, Nicola Persad, Claire Brown; *New Cross Hospital (15),* D Morgan, Karla Preece; *Norfolk and Norwich University Hospital (17),* Ingrid Watmough, Esther Thomas, Niki Wyatt; *North Manchester General Hospital (29),* H Finneran, Stephen Duberley; *Norwich Community Hospital (9),* Ingrid Watmough, Esther Thomas, Niki Wyatt; *Pendle Community Hospital (15),* M Goorah, A Sangster; *Royal Blackburn Hospital (8),* M Goorah, A Sangster; *Royal Bournemouth Hospital (9),* Anna Orpen, Catherine Ovington; *Royal Cornwall Hospital (19),* F Harrington, G Courtauld, Christine Schofield, Ali James; *Royal Devon and Exeter Hospital (18),* Leigh Barron, Nicola Wedge; *Royal Preston Hospital (15),* Lorna Fazackerley, Stephen Duberley, Christina Gilmour, Bindu Gregary; *Singleton Hospital (45),* Helen Goldring; *Southampton General Hospital (13),* G Durward, Vanessa Pressly; *Southlands Hospital (8),* Rajen Patel, Carol Simmons, Angela Dunne; *Southport and Formby District General Hospital (14),* Helen Terrett; *Stoke Mandeville Hospital (15),* Susan Buttfield; *St Peters Hospital (29),* Emma Young; *Sunderland Royal Hospital (2),* Janice O'Connell; *Tyrone County Hospital (5),* Celia Diver-Hall; *Walsall Manor Hospital (45),* Jo McCormack, Karla Preece, Steve Hurdowar; *Warrington Hospital (9),* O Otaiku, Lynne Connell; *West Park Rehabilitation Hospital (14),* D Morgan, Karla Preece; *Whiston Hospital (20),* Sharon Dealing; *Williton Hospital (18),* Libby Caudwell, Sarah Edwards; *Worthing Hospital (6),* Rajen Patel, Carol Simmons, Angela Dunne; *University Hospital, Coventry (18),* C Randall, Lucy Aldridge, Martine Pritchard; *University Hospital of Hartlepool (34),* David Bruce, Susan Crawford; *University Hospital of North Durham (0),* Cath Huntley; *University Hospital of North Tees (17),* Helen Skinner, Susan Crawford

Location of services – categorised by UK Strategic Health Authority (geographical areas).

	Control (n=16)	Intervention (n=16)	Total (n=32)
**England:**			
North East	1	2	3
North West	2	4	6
East Midlands	1	0	1
West Midlands	2	1	3
South East Coast	2	1	3
South Central	1	0	1
South West	3	3	6
East	1	1	2
Scotland	1	1	2
Northern Ireland	2	1	3
Wales	0	2	2

The LoTS care system of care (intervention)

The LoTS care system of care was developed (2000-2006) in keeping with the MRC Framework for the development and evaluation of complex interventions[1] and was based on systematic reviews and synthesis of qualitative and quantitative literature, national surveys, interviews with patients and carers and feasibility testing. Through these procedures a structured and systematic approach to patient assessment which was based on the problems that patients and carers themselves had identified as of central importance was developed.

In order to identify treatment algorithms, in collaboration with information specialists at the University of Leeds, a hierarchical comprehensive structured protocol for identifying evidence in each of the 15 problem areas was developed. This protocol included identifying relevant stroke specific and problem specific guidelines, meta-analyses and systematic reviews, and if necessary individual randomised controlled trials. Two researchers independently reviewed all outputs. Guidelines, reviews and papers identified for inclusion were assessed for quality using standard tools. Drafted treatment algorithms were peer reviewed by external experts, prior to compilation in the manual developed to support our newly developed system of care.

The resulting LoTS care stroke system of care manual comprises: patient and carer structured assessment representing the identified problem areas linked to a reference guide and treatment algorithm; a directory of service information; and a selection of validated assessment scales for specific areas such as depression and cognitive impairment are included as appendices in the manual.

A Care Plan was developed to be completed for each patient (and if relevant, carer) following each contact which included patient details, the structured assessment and related prompt questions, patient and carer goal and action plans.

An optional checklist detailing the content of the assessment to be given to patients beforehand was also developed (see below). Thus a manualised structured system for longer-term stroke care that is comprehensive (encompasses all areas of potential concern to patient and carer) but individualised (patient specific action plans constructed) was created.

1. Craig P, Dieppe P, Macintyre S, Michie S, I Nazareth I, Petticrew M, Developing and evaluating complex interventions: new guidance. London: Medical Research Council; 2008.

CLIENT CHECKLIST

These are the main areas that will be addressed during your assessment.
If you wish, you can make a note of any concerns or queries on this sheet as a reminder.

Communication - Information needs about your stroke and who to contact.

Medicine – Any problems relating to your medication.

Pain – Any problems with pain and current treatment.

Mobility/falls – Getting around inside and outside your home and fear of falling.

Personal hygiene – Washing/bathing, cutting toenails, dressing, dental treatment.

Shopping/meal preparation – Getting to the shops, getting a balanced diet, making meals.

House & home – Doing housework, gardening, general repairs or finding a new home.

Memory – Difficulty remembering things or concentrating.

Transport – Getting back to driving or using private / public transport.

Finances & benefits – Organising bills payments, entitlements, council rebates, working.

Continence – Need for pads etc., further assessments, advice.

Sexual relations – Need for advice on changes to your intimate life.

Mood (patient) – Feeling down, anxious or worried.

Social life (patient) - Day-time interests and hobbies, getting to see others, religious practice, relationship problems, having a holiday.

Mood (partner/relative/friend) – Feeling down, anxious, worried, coping with your caring role in any of the above areas e.g. paying bills, house and home, patient's personal hygiene.

Social (partner/relative/friend) – Having a break, arranging a holiday, relationship problems, working.

Any other problems?

Patients structured assessment (as represented in the Care Plan see below)

**Transfer of Care - Reference Guide 1**<br>Has a discharge plan been prepared by the hospital?<br>Have you had a home visit? What was the outcome?<br>(*Discuss issues around knowledge/understanding of care plan, involvement, falls management, information sources*)
**Communication & Information - Reference Guide 2**<br>Have you been given clear information about your condition, treatment and services availaable?
**Medicines & General Health - Reference Guide 3**<br>Do you have any problems with your medication (*side-effects, drug cocktail, non-prescription drugs*)?<br>Do you have any problems taking your medication?<br>Do you have any other health problems?<br>Are these being reviewed?
**Pain - Reference Guide 4**<br>Do you have any pain? Is the pain being treated? Is the treatment helping?
**Mobility/Falls - Reference Guide 5**<br>Can you get around indoors?<br>Can you get out of your house?<br>How confident are you about carrying out various daily activities without falling?<br>How do you feel about your recovery so far?<br>*(Discuss patterns of physical and emotional recovery)*
**Personal Hygiene & Dressing - Reference Guide 6**<br>Are you having any difficulty with personal care like washing, cutting your nails, oral hygiene or dressing?<br>Do you ever need help?
**Shopping & Meal Preparation - Reference Guide 7**<br>Can you prepare your own meals?<br>Can you go shopping?<br>Are you getting the right food?

**House & Home - Reference Guide 8**<br>Can you do your housework?<br>Does anyone help?<br>Do you have any problems with your accommodation?
**Cognition - Reference Guide 9**<br>Do you often have a problem remembering things that happened recently?<br>Do you often forget where you have put things?<br>Do you find it difficult to concentrate?
**Driving & General Transport - Reference Guide 10**<br>Do you have trouble with transport?
**Finances & Benefits - Reference Guide 11**<br>Do you have any difficulty managing your money?<br>Are you able to pay your bills?<br>Are you receiving all the benefits you are entitled to? (*Use a benefits checklist*)
**Continence - Reference Guide 12**<br>Do you ever have accidents with your bladder/bowel? How is this being managed?
**Sexual Functioning - Reference Guide 13**<br>Some people after stroke experience sexual problems such as lack of interest or practical difficulties. Have you experienced any changes sexually since your stroke?
**Patient Mood - Reference Guide 14**<br>Have you recently felt very sad or fed up?<br>Have you felt anxious, frightened or worried?<br>Do you have a partner, relative or friend you feel close to? Do you get on well? Can you talk about your worries or problems with them?
**Patient Social Needs - Reference Guide 15**<br>How do you spend your day?<br>Do you have enough to do?<br>Are you happy with your social life?<br>Do you wish you had more social contact with others?<br>(*Discuss work as appropriate, changes in role, self image, and relationships.*)
**Other** Are you having any other problems relating to your stroke?

The manual is available from the corresponding author: a.forster@leeds.ac.uk

Sample (contact 1) of the Care Plan

Contact 1 *(Community-based)*

Not including telephone calls to make appointments

Date: Day / Month / Year   Carer involved? Y N n/a   Type of contact: ☐ Face-to-face ☐ Telephone

Your name ____   Location: ☐ Home ☐ Clinic ☐ Other, specify: ____

Profession *e.g. nurse* ____ NHS Band ____

☐ Client checklist provided in advance of the assessment

Carer information

Does the patient have a carer?
Defined as: main informal carer who provides the patient with practical support a minimum of once per week

☐ Yes
☐ No

Was a carer assessment completed?

☐ Yes
☐ No → Reason assessment not completed:
- ☐ Carer assessments routinely provided by other services
- ☐ Carer not present
- ☐ Carer declined
- ☐ Not offered *(reason)*:
- ☐ Other *(reason)*:

☐ Patient does not have a carer (as per definition) but has care package provided ____ *Number of times a day/week*

Patient assessment questions

Remember to ask all of the questions *If there is a problem but it is not going to be addressed now, please tick the problem box and record in the action plan that it will be revisited next time.*	Response		If NOT asked: *indicate why*	
	No problem. No further action.	Problem. Please go to ACTION PLAN	Already addressed	Other. Please explain in notes section.
**Transfer of Care: Reference guide 1**				
Has a discharge plan been prepared by the hospital? Have you had a home visit? What was the outcome? *Discuss issues around knowledge and understanding of their care plan, their involvement, falls management and sources of information.*	☐	☐	☐	☐
**Communication & Information: Reference guide 2**				
Have you been given clear information about your condition, treatment and services available?	☐	☐	☐	☐

Patient's Name ____ Patient's D.O.B Day / Month / Year NHS number ____

6

Contact 1

Remember to ask all of the questions *If there is a problem but it is not going to be addressed now, please tick the problem box and record in the action plan that it will be revisited next time.*	Response		If NOT asked: *indicate why*	
	No problem. No further action.	Problem. Please go to ACTION PLAN	Already addressed	Other. Please explain in notes section.
**Medicines & General Health: Reference guide 3**				
Do you have any problems with your medication? *For example, side-effects, drug cocktail, non-prescription drugs.* Is medication being regularly reviewed by a doctor/nurse? *Medication can be recorded on page 4.*	☐	☐	☐	☐
Do you have any problems taking your medication? *For example, opening packets, remembering, swallowing (check for swallowing assessment).*	☐	☐	☐	☐
Do you have any other health problems? *Ask about smoking cessation, alcohol consumption, diet and weight loss.* Check if other health problems are being regularly reviewed.	☐	☐	☐	☐
**Pain: Reference guide 4** *(assessment scale available)*				
Do you have any pain? Is the pain being treated? Is the treatment helping?	☐	☐	☐	☐
**Mobility / Falls: Reference guide 5** *(assessment scale available)*				
Can you get around indoors?	☐	☐	☐	☐
Can you get out of your house?	☐	☐	☐	☐
How confident are you about carrying out various daily activities without falling?	☐	☐	☐	☐
How do you feel about your recovery so far? *Discuss patterns of physical and emotional recovery*	☐	☐	☐	☐

Patient's Name ____ Patient's D.O.B Day / Month / Year NHS number ____

7

Contact 1 patient assessment questions *(continued)*

Remember to ask all of the questions *If there is a problem but it is not going to be addressed now, please tick the problem box and record in the action plan that it will be revisited next time.*	Response: No problem. No further action.	Response: Problem. Please go to ACTION PLAN	If NOT asked: *indicate why* Already addressed	If NOT asked: Other. Please explain in notes section.
**Personal Hygiene & Dressing: Reference guide 6**				
Are you having any difficulty with personal care like washing, cutting your nails, oral hygiene or dressing? Do you ever need help? *If applicable, discuss satisfaction with homecare services (e.g. dressing, bathing).*	☐	☐	☐	☐
**Shopping & Meal Preparation: Reference guide 7**				
Can you prepare your own meals?	☐	☐	☐	☐
Can you go shopping?	☐	☐	☐	☐
Are you getting the right food?	☐	☐	☐	☐
**House & Home: Reference guide 8** *(assessment scale available)*				
Can you do your housework? Does anyone help you with your housework?	☐	☐	☐	☐
Do you have any problems with your accommodation? *If applicable, discuss satisfaction with homecare services (e.g. cleaning, shopping and laundry).*	☐	☐	☐	☐
**Cognition: Reference guide 9** *(assessment scale available)*				
Do you often have a problem remembering things that happened recently? Do you often forget where you have put things? Do you find it difficult to concentrate?	☐	☐	☐	☐

Patient's Name | Patient's D.O.B (Day Month Year) | NHS number:

Contact 1

Remember to ask all of the questions *If there is a problem but it is not going to be addressed now, please tick the problem box and record in the action plan that it will be revisited next time.*	Response: No problem. No further action.	Response: Problem. Please go to ACTION PLAN	If NOT asked: *indicate why* Already addressed	If NOT asked: Other. Please explain in notes section.
**Driving & General Transport: Reference guide 10** *(assessment scale available)*				
Do you have trouble with transport?	☐	☐	☐	☐
**Finances & Benefits: Reference guide 11**				
Do you have any difficulty managing your money?	☐	☐	☐	☐
Are you able to pay your bills?	☐	☐	☐	☐
Are you receiving all the benefits you are entitled to? *Use a benefits checklist.*	☐	☐	☐	☐
**Continence: Reference guide 12** *(assessment scale available)*				
Do you ever have accidents with your bladder/bowel? How is this being managed?	☐	☐	☐	☐
**Sexual Functioning: Reference guide 13** *(assessment scale available)*				
Some people after a stroke experience sexual problems such as lack of interest or practical difficulties. Have you experienced any changes sexually since your stroke?	☐	☐	☐	☐

Patient's Name | Patient's D.O.B (Day Month Year) | NHS number:

Contact 1 patient assessment questions *(continued)*

Remember to ask all of the questions *If there is a problem but it is not going to be addressed now, please tick the problem box and record in the action plan that it will be revisited next time.*	Response		If NOT asked: *indicate why*	
	No problem. No further action	Problem. Please go to ACTION PLAN	Already addressed	Other. Please explain in notes section
**Patient Mood: Reference guide 14** *(assessment scale available)*				
Have you recently felt very sad or fed up?	☐	☐	☐	☐
Have you felt anxious, frightened or worried? *Do you find it difficult to control your emotions? Discuss as appropriate frustration and irritability.* Have you been offered or are you having any treatment?	☐	☐	☐	☐
Do you have a partner, relative or friend you feel close to? Do you get on well? Can you talk about your worries or problems with them? *Discuss feelings of burden.*	☐	☐	☐	☐
**Patient Social Needs: Reference guide 15**				
How do you spend your day? Do you have enough to do? Are you happy with your social life? Do you wish you had more social contact with others? *Discuss work as appropriate, changes in role, relationships, personality, attitude and confidence. Enquire about self image and lack of energy. Discuss patient's perception of life since the stroke as compared with pre-stroke, and any thoughts about the future.*	☐	☐	☐	☐
**Other**				
Are you having any other problems relating to your stroke? *For example, swallowing, speech & language difficulties, sleeping, vision, numbness, oral health? Enquire if the patient has set self goals and what they are. Ask about their expectations and general patterns of physical and emotional recovery.* Discuss motivation and how much control they feel they have in their recovery.	☐	☐	☐	☐

Patient's Name | Patient's D.O.B (Day Month Year) | NHS number:

Notes

Contact 1

Patient's Name | Patient's D.O.B (Day Month Year) | NHS number:

Contact 1 goal and action planner

Contact 1

Client information: During your assessment you will plan and agree some goals to work towards or actions to take with your stroke care co-ordinator (SCC). The actions and goals may be set for you or for your SCC. After you have attempted your goals and actions, record the outcome below.

Duration of assessment contact (time spent with patient) [Hours : Minutes] (hours/mins)

Time spent discussing patient in MDT – *please complete table on back page.*

Time for additional note writing [Hours : Minutes] (hours/mins)

Problem <br>***(Please number problems)***	***Assessment question(s)/ Related reference guide(s) number***	***Goal or action***	***Patient / SCC***	***Date set***	***Date review***	***Review of outcome*** ***Please write*** ***a: Goal achieved,*** ***b: Nearly there but extra guidance required,*** ***c: Struggling with goal – To be reviewed in next contact***	***Duration spent on each SCC action between date set and date review. (HH:MM)*** *(exclude assessment contact time, include phone calls etc.)*
1. Difficulty going shopping because of parking and walking problems	5, 7 & 10	Obtain Blue badge	Patient	01.02.2009	01.03.2009	a. Blue Badge obtained	20 mins
**LoTS care trial team use only:** Total number of problems identified [ ]	Reference guides used: 1 2 3 4 5 6 7 8 9 10 11 12 13 14 15 Other	Total number of goals/actions: [ ]	Total number of patient goals/actions: [ ]			Total number achieved: [ ]	Total duration: [Hours : Minutes]

Training in the LoTS care system of care

Training in the delivery of the system of care was provided for each of the SCC services randomised to the intervention through two centrally based Royal College of Nursing (England) accredited training days approximately one month apart. Because of the nature of a cluster trial the SCCs randomised to the intervention were unaware of what the new service model consisted of prior to these training days. The system of care was well received when presented by the trial team demonstrating 'face validity' for the intervention.

In the first training day the principles of the intervention were presented as described below:

- Patient-centred (comprehensive coverage of problems identified by patients and carers)
- Provide assessment areas (checklist) before assessment whenever possible
- Ask all assessment questions
- Keep accurate records
- Problem solving approach with collaborative goal setting
- Follow-up on actions
- Review goals
- Non prescriptive – individual creativity
- According to local services / resources
- Within patient's own environment wherever possible
- Timing / duration of intervention (according to national recommendations)
- Flexible approach to carer assessment

Practical issues of implementation were addressed, specifically, design of the care plans and manual. A workshop was led by Professor Allan House on assessment and problem solving techniques.

The content of the second training day was informed by the needs of the SCCs. Thus a lecture on pain management was provided (by Professor Tony Rudd or Dr Peter Wanklyn) and discussion on benefits was led by a colleague from the Department for Work and Pensions. The format and layout of the Care Plan was further reviewed and refined following the SCCs' feedback. The intent was to ensure that the paperwork captured all information that they might require to deliver their service, and therefore replace the current documentation.
The training was supported by a CD of the training day. After the second training day, the SCCs were asked to use the Care Plan for all patient contacts (and carers if carer assessments were performed).The SCCs implemented the new system of care in their service over four to six months providing sufficient time for the implementation of the system of care into standard practice, when they were comfortable with implementation (demonstrated through review of completed care plans by the trial team) trial recruitment began.

Full details of the development of the LoTS care system of care is available in:
Forster A, Mellish K, , Farrin A, Bhakta B, Murray J, Patel A, House A, Hewison J, Knapp M, Breen R, Chapman K, Holloway I, Hawkins R, Shannon R, Nixon J, Jowett A, Horton M, Alvarado N, Anwar S, Tennant A, Godfrey M, Young J. Development and evaluation of interventions and tools to improve patient and carer centred outcomes in longer-term stroke care (LoTS care) and exploration of adjustment post stroke. NIHR Journals Library. For further information see: http://www.journalslibrary.nihr.ac.uk/pgfar/volume-2/issue-6#abstract December 2014. Access date April 2015.

Summary of potential barriers and facilitators to intervention delivery

The system of care was developed in 2000-2006. Although review of patients' post-stroke needs is recommended policy, no systematic approach had previously been developed for routine monitoring, problem identification and co-ordination of services to assist stroke patients and their families as they continue to recover from their stroke and make life adjustments to its consequences. The LoTS care system of care was based on a systematic review and synthesis of the available qualitative literature reporting interviews with stroke patients and carers in which longer-term stroke-related issues were discussed.[1] A complementary review of quantitative stroke surveys assessed the prevalence of these problem areas.[2] To confirm content validity, emerging findings were checked and refined by stroke patients and carers in individual interviews and in focus group.[3] This approach ensured that the system of care is targeted at the most common stroke-related problems of central importance to stroke patients and their carers. Intervention implementation was informed by policy recommendations and a national survey.

Facilitators to intervention delivery within this trial evaluation included:

- All Stroke Care Co-ordinators (SCCs) randomised to deliver the intervention recognised the importance of the work and relevance of the content of the system of care (details of the intervention were not divulged until after site randomisation).
- The intervention was supported by a detailed manual which included 16 assessment questions (patient) and 13 questions (carer) representing the identified patient/carer problem areas, linked to reference guides containing educational text with algorithms of evidenced-based treatment options. The SCCs were also provided with a 'Care Plan' which included the assessment questions and a goal and action planner to be completed following each contact (patients and carers).
- Intervention SCCs were actively engaged in finalising the intervention materials. Specifically the Care Plan was adapted to capture all information that they might require to deliver their service, and therefore replace the current documentation. Thus the LoTScare Care Plan became embedded in routine practice rather than an additional component.
- The manual was recognised as a useful tool to promote multidisciplinary working
- The SCCS had opportunity to practice the new intervention between the two training days which enabled discussion with the trial team and their peers at the second training day.
- The training was delivered by eminent stroke clinicians/researchers enhancing credibility.
- The training was responsive to specific needs (for example, specific training was provided on pain management and benefits available).
- The training was supported by a CD of the training day, which could be accessed later.
- Participant recruitment was not opened until all parties were satisfied that the system of care was being delivered,

Potential barriers to intervention delivery:

- Some SCCs left and were replaced during the trial and in services where there were multidisciplinary teams all SCCs did not necessarily attend the training. The intent was that the training would be cascaded down by staff who attended the training days

to other staff in the team. It may be that this commonly used 'cascade' method was not as effective as we would have wished.

- It may be that in this service model the notion of comprehensive holistic assessment becomes dissipated by individual allied health professionals delivering their single discipline input, for example, therapists prioritising mobility problems.
- Whilst the number of assessment contacts was not prescribed, a key principle of the intervention is that goals are reviewed at subsequent contacts, which will necessarily be limited if the number of contacts are small.
- Despite regular multidisciplinary team meetings the original Care Plan assessment had not always been re-visited to check that actions had been undertaken and goals reached.
- Although all of the reference guides are evidence-based, the evidence points to more effective interventions for certain problems than for others, thereby weakening the overall effectiveness of this complex intervention.
- The challenge in pragmatic multicentre trial evaluation in rehabilitation is to provide some guidance on the intervention to capture the main features, enhance external validity and improve generalisability whilst making it sufficiently flexible for it to be acceptable to staff and deliverable in a range of service models.[4] Our approach was to manualise the intervention supported by training days with an opportunity to practice delivery prior to the start of patient recruitment. The Care Plan was well completed, but we were perhaps less successful in changing the behaviours and mind set of the SCCs. Further work should explore how to embed behaviour change techniques in both intervention training and delivery and in the intervention itself.

References

1. Murray J, Ashworth R, Forster A, Young J. Developing a primary care-based stroke service: a review of the qualitative literature. *Br J Gen Pract*. 2003;53:137-42.
2. Murray J, Young J, Forster A, Ashworth R. Developing a primary care-based stroke model: the prevalence of longer-term problems experienced by patients and carers. *Br J Gen Pract* 2003;53:803-7.
3. Murray J. The development of a primary care-based model for after stroke care. [PhD Thesis]. Leeds: University of Leeds; 2007.
4. Hawe P, Shiel A, Riley T. Complex interventions: how "out of control" can a randomised controlled trial be? *BMJ* 2004; 328: 1561–3.

Brief overview of differences between treatment arms

Considerable detail was collected on the participating services before, during and after the trial. This included a survey of existing practices prior to enrolling in the trial and semi-structured interviews with all participating Stroke Care Co-ordinators (SCCs) at the onset of the trial in which they described their service and client group. These interviews were repeated mid-way through recruitment and after 12 months follow-up. Through these interviews they described their service and client group (including any changes).

Prior to commencement of the trial none of the SCCs used or had access to an assessment schedule that was specifically structured round the longer-term problems of stroke patients and their carers, or had access to a manual of evidenced-based treatment algorithms linked to these assessment questions.

The interviews before, during and after the trial demonstrated there were clear differences between the control and intervention arms.

- Intervention SCCs were provided with specific training which included techniques of the problem solving approach (supported by a CD which they could access later) which was not provided to the Control SCCs.
- Intervention SCCs provided patients with the LoTS care checklist prior to assessment, whereas no control SCCs mentioned an equivalent checklist in their service.
- None of the Control site SCCs had an assessment schedule created around the identified problems of stroke patients and their carers.
- None of the Control site SCCs had a purposely designed Care Plan which included the assessment questions and a goal and action planner to be completed following each contact (patients and carers).
- None of the Control site SCCs used or had access to a manual of evidenced-based treatment.
- Although control SCCs intended to provide a fully holistic initial assessment, it is not clear from interviews whether they actually did, whereas the SCCs in the intervention arm appeared to provide a holistic assessment more consistently.

SUPPLEMENTAL TABLES

Response rates for patients and carer outcomes

Response rates for patient reported outcomes at six months were: 75.2% (300/399) in control and 77.3% (310/401) in intervention and at 12 months 67.2% (268/399) in control and 70.1% (281/401) in intervention. Response rates for carer reported outcomes at six months were: 88.0% (88/100) in control and 82.4% (89/108) in intervention and at 12 months 71.0% (71/100) in control and 67.6% (73/108) in intervention.

Table I Carer baseline demographic details

Carer baseline demographic details		
**Carer**	**Control (n=100)**	**Intervention (n=108)**
Age – Mean (SD)	61.4 (14.07)	61.0 (15.02)
Sex: Male – N (%)	32 (32.0)	35 (32.4)
Ethnicity: White – N (%)	99 (99.0)	105 (97.2)
Formal education – N (%)	97 (97.0)	103 (95.4)
If formal education: Left education at 16 or less - N (%)	68 (70.1)	76 (73.8)
Carer - patient relationship – N (%)		
Partner	67 (67.0)	70 (64.8)
Daughter / son	29 (29.0)	33 (30.6)
Other	4 (4.0)	5 (4.6)
Living with patient post stroke – N (%)	78 (78.0)	85 (78.7)

LUNS Questionnaire outcomes

The number and types of unmet needs reported (LUNS questionnaire) were similar for the control and intervention groups at six and 12 months. A median of 2.5 unmet needs was reported; the prevalence of individual unmet needs ranged from 2% to 51% of those completing the questionnaire.

Table II LUNS summaries – number of unmet needs for patients at 6 months

LUNS summaries<br>Number of unmet needs for patients at 6 months			
	**Control (n=399)**	**Intervention (n=401)**	**Total (n=800)**
**N of unmet needs at 6 months**			
0	57 (14.3%)	43 (10.7%)	100 (12.5%)
1	46 (11.5%)	46 (11.5%)	92 (11.5%)
2	44 (11.0%)	41 (10.2%)	85 (10.6%)
3	25 (6.3%)	39 (9.7%)	64 (8.0%)
4	28 (7.0%)	31 (7.7%)	59 (7.4%)
5	15 (3.8%)	16 (4.0%)	31 (3.9%)
6	18 (4.5%)	17 (4.2%)	35 (4.4%)
7	15 (3.8%)	18 (4.5%)	33 (4.1%)
8	13 (3.3%)	17 (4.2%)	30 (3.8%)
9	8 (2.0%)	8 (2.0%)	16 (2.0%)
10	3 (0.8%)	10 (2.5%)	13 (1.6%)
11	3 (0.8%)	5 (1.2%)	8 (1.0%)
12	7 (1.8%)	1 (0.2%)	8 (1.0%)
13	4 (1.0%)	7 (1.7%)	11 (1.4%)
14	2 (0.5%)	1 (0.2%)	3 (0.4%)
15	1 (0.3%)	1 (0.2%)	2 (0.3%)
16	0 (0.0%)	1 (0.2%)	1 (0.1%)
17	1 (0.3%)	0 (0.0%)	1 (0.1%)
18	0 (0.0%)	1 (0.2%)	1 (0.1%)
Missing	109 (27.3%)	98 (24.4%)	207 (25.9%)

Table III LUNS summaries – number of unmet needs for patients at 12 months

LUNS summaries<br>Number of unmet needs for patients at 12 months			
	**Control (n=399)**	**Interventio n (n=401)**	**Total (n=800)**
**N of unmet needs at 12 months**			
0	62 (15.5%)	62 (15.5%)	124 (15.5%)
1	35 (8.8%)	36 (9.0%)	71 (8.9%)
2	34 (8.5%)	43 (10.7%)	77 (9.6%)
3	29 (7.3%)	31 (7.7%)	60 (7.5%)
4	20 (5.0%)	28 (7.0%)	48 (6.0%)
5	21 (5.3%)	11 (2.7%)	32 (4.0%)
6	11 (2.8%)	12 (3.0%)	23 (2.9%)
7	12 (3.0%)	19 (4.7%)	31 (3.9%)
8	10 (2.5%)	8 (2.0%)	18 (2.3%)
9	10 (2.5%)	7 (1.7%)	17 (2.1%)
10	6 (1.5%)	7 (1.7%)	13 (1.6%)
11	4 (1.0%)	3 (0.7%)	7 (0.9%)
12	2 (0.5%)	6 (1.5%)	8 (1.0%)
13	3 (0.8%)	2 (0.5%)	5 (0.6%)
14	1 (0.3%)	1 (0.2%)	2 (0.3%)
15	1 (0.3%)	0 (0.0%)	1 (0.1%)
18	1 (0.3%)	0 (0.0%)	1 (0.1%)
21	0 (0.0%)	1 (0.2%)	1 (0.1%)
Missing	137 (34.3%)	124 (30.9%)	261 (32.6%)

Figure I Prevalence of individual unmet needs at 6 months and 12 months (out of those who returned completed LUNS questionnaire)

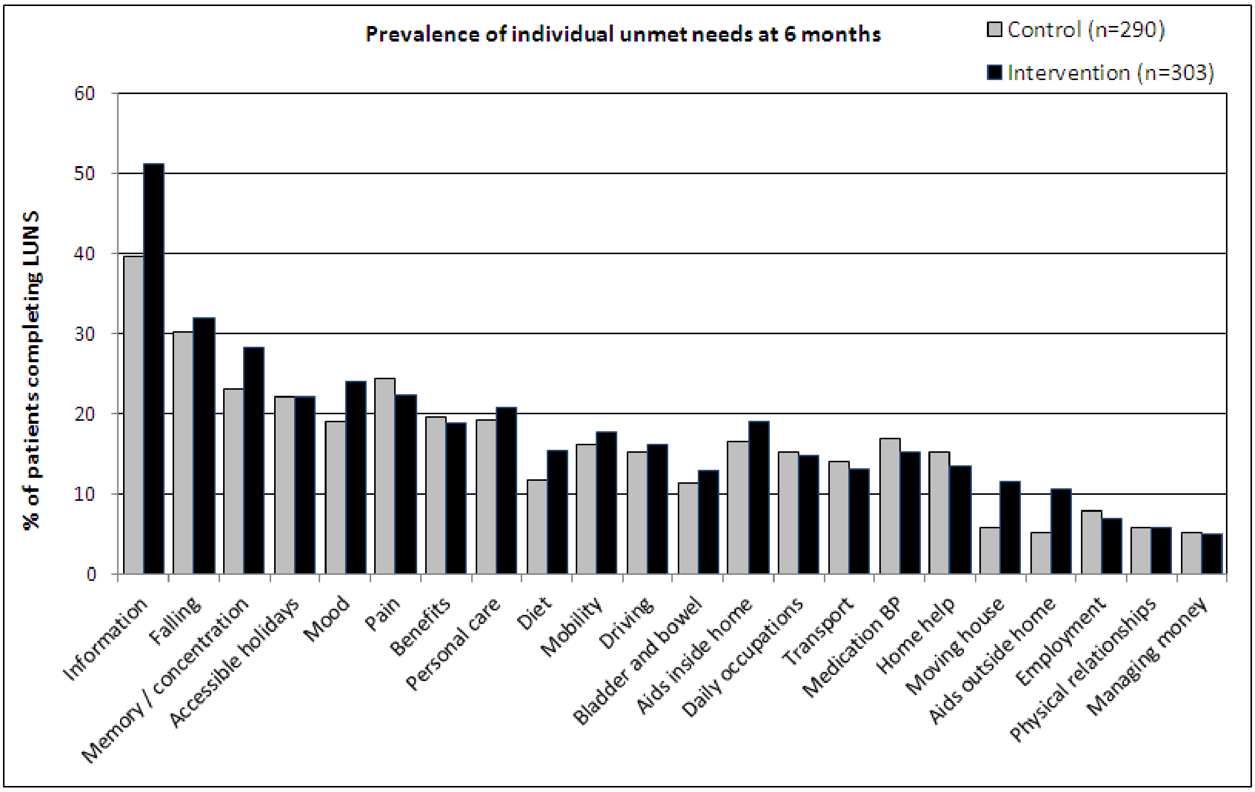

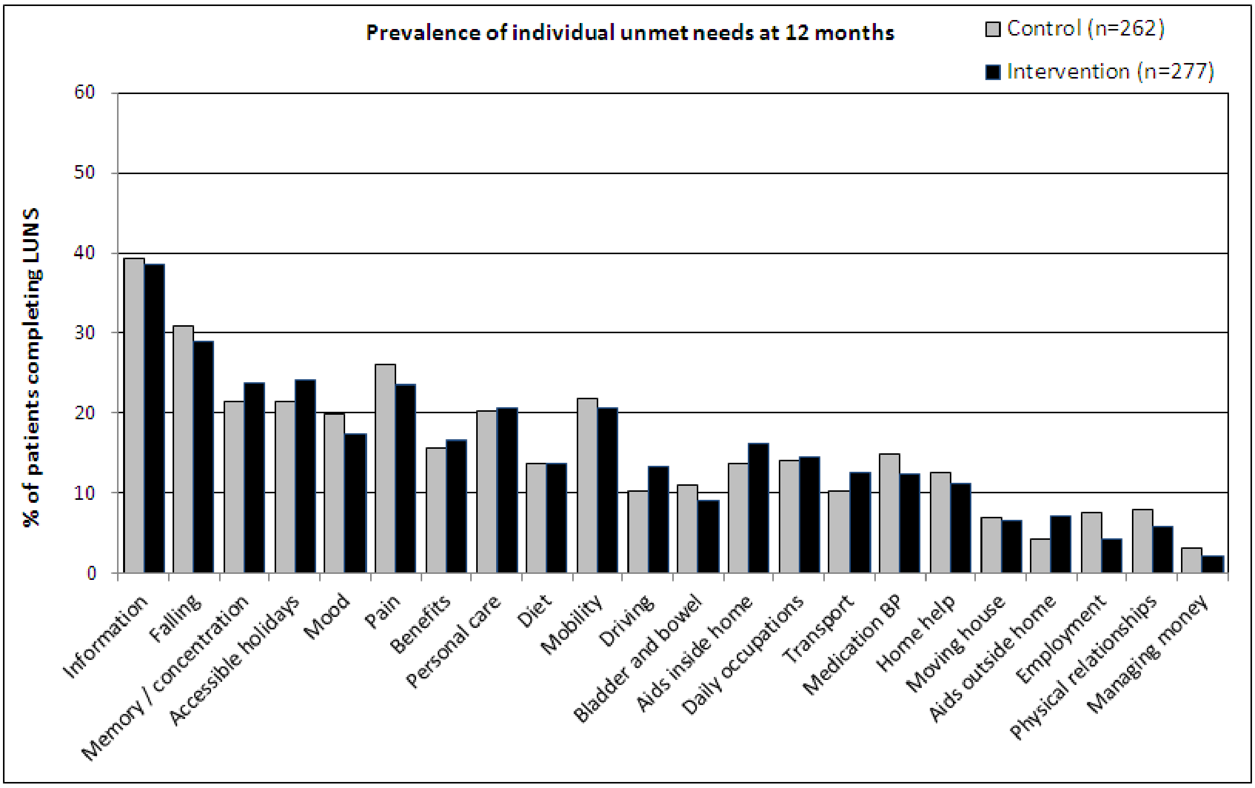

Table IV Summaries of patient deaths

	Patients deaths		
	**Control (n=399)**	**Intervention (n=401)**	**Total (n=800)**
**Patient deaths**			
6 months timepoint	27 (6.8%)	31 (7.7%)	58 (7.3%)
12 months timepoint	5 (1.3%)	4 (1.0%)	9 (1.1%)
After 12 months follow-up*	2 (0.5%)	2 (0.5%)	4 (0.5%)
**Overall deaths**	32 (8.0%)	35 (8.7%)	67 (8.4%)

*Not included in the overall deaths as reported after the follow up period

Table V Treatment on an emergency outpatient basis and hospital admissions at 6 months

6 month researcher follow up summary			
	**Control (n=399)**	**Intervention (n=401)**	**Total (n=800)**
**Follow-up form completed N (%)**			
Yes	395 (99.0%)	400 (99.8%)	795 (99.4%)
Withdrawn	4 (1.0%)	1 (0.2%)	5 (0.6%)
**Patient visited A&E in past six months N (%)**			
Yes	105 (26.3%)	113 (28.2%)	218 (27.3%)
No	290 (72.7%)	261 (65.1%)	551 (68.9%)
Missing	4 (1.0%)	27 (6.7%)	31 (3.9%)
**Number of A&E visits per patient**			
Mean (SD)	0.4 (0.95)	0.4 (0.83)	0.4 (0.89)
Median (Range)	0.0 (0.0, 8.0)	0.0 (0.0, 7.0)	0.0 (0.0, 8.0)
**Patient admitted to hospital overnight in past 6 months N (%)**			
Yes	113 (28.3%)	97 (24.2%)	210 (26.3%)
No	282 (70.7%)	303 (75.6%)	585 (73.1%)
Missing	4 (1.0%)	1 (0.2%)	5 (0.6%)
**Number of hospital admissions**			
Mean (SD)	0.4 (0.93)	0.3 (0.66)	0.4 (0.81)
Median (Range)	0.0 (0.0, 10.0)	0.0 (0.0, 5.0)	0.0 (0.0, 10.0)
**Number of patients that died during hospital admission**			
	13 (3.3%)	10 (2.5%)	23 (2.9%)
**Number of patients that spent time in a specialist care unit**			
	8 (2.0%)	4 (1.0%)	12 (1.5%)
**Number of times spent in a specialist care unit**			
Mean (SD)	0.0 (0.20)	0.0 (0.13)	0.0 (0.17)
Median (Range)	0.0 (0.0, 3.0)	0.0 (0.0, 2.0)	0.0 (0.0, 3.0)
**Time spent in a specialist care unit (days)**			
Mean (SD)	0.2 (1.36)	0.0 (0.63)	0.1 (1.06)
Median (Range)	0.0 (0.0, 20.0)	0.0 (0.0, 11.0)	0.0 (0.0, 20.0)

Table VI Treatment on an emergency outpatient basis and hospital admissions at 12 months

12 month researcher follow up summary	Control (n=399)	Intervention (n=401)	Total (n=800)
**Follow-up form completed N (%)**			
Yes	374 (93.7%)	374 (93.3%)	748 (93.5%)
Died	21 (5.3%)	26 (6.5%)	47 (5.9%)
Withdrawn	4 (1.0%)	1 (0.2%)	5 (0.6%)
**Patient visited A&E in past six months N (%)**			
Yes	71 (17.8%)	77 (19.2%)	148 (18.5%)
No	303 (75.9%)	275 (68.6%)	578 (72.3%)
Missing	25 (6.3%)	49 (12.2%)	74 (9.3%)
**Number of A&E visits per patient**			
Mean (SD)	0.3 (1.17)	0.3 (0.66)	0.3 (0.95)
Median (Range)	0.0 (0.0, 19.0)	0.0 (0.0, 4.0)	0.0 (0.0, 19.0)
**Patient admitted to hospital overnight in past 6 months N (%)**			
Yes	74 (18.5%)	77 (19.2%)	151 (18.9%)
No	300 (75.2%)	297 (74.1%)	597 (74.6%)
Missing	25 (6.3%)	27 (6.7%)	52 (6.5%)
**Number of hospital admissions**			
Mean (SD)	0.3 (0.73)	0.3 (0.68)	0.3 (0.71)
Median (Range)	0.0 (0.0, 5.0)	0.0 (0.0, 4.0)	0.0 (0.0, 5.0)
**Number of patients that died during hospital admission**			
	3 (0.8%)	5 (1.2%)	8 (1.0%)
**Number of patients that spent time in a specialist care unit**			
	2 (0.5%)	4 (1.0%)	6 (0.8%)
**Number of times spent in a specialist care unit**			
Mean (SD)	0.0 (0.07)	0.0 (0.13)	0.0 (0.11)
Median (Range)	0.0 (0.0, 1.0)	0.0 (0.0, 2.0)	0.0 (0.0, 2.0)
**Time spent in a specialist care unit (days)**			
Mean (SD)	0.0 (0.29)	0.0 (0.56)	0.0 (0.45)
Median (Range)	0.0 (0.0, 5.0)	0.0 (0.0, 10.0)	0.0 (0.0, 10.0)

Table VII: Patients' unadjusted questionnaire scores at different time points by arm

Questionnaire	Baseline Control Mean (SD) N	Baseline Intervention Mean (SD) N	6 months Control Mean (SD) N	6 months Intervention Mean (SD) N	12 months Control Mean (SD) N	12 months Intervention Mean (SD) N
Barthel Index pre-stroke	19.2 (1.98) 399	19.2 (2.22) 401	N/A	N/A	N/A	N/A
Barthel	15.2 (4.48) 398	14.4 (5.06) 401	16.2 (4.24) 296	15.5 (4.48) 307	16.3 (3.91) 266	15.7 (4.26) 282
GHQ12	15.2 (7.26) 396	15.9 (7.03) 397	15.3 (7.32) 305	16.2 (7.17) 318	14.5 (6.78) 268	14.3 (6.87) 281
EQ5D	0.56 (0.340) 382	0.51 (0.378) 381	0.61 (0.339) 288	0.56 (0.322) 301	0.61 (0.316) 259	0.54 (0.318) 270
FAI	28.1 (9.71) 398	27.8 (9.70) 399	20.3 (11.41) 293	18.4 (11.27) 304	21.5 (11.30) 266	18.8 (11.53) 281

Patients' unadjusted questionnaire scores at different time points by arm

Table VIII Patient adjusted questionnaire scores at 6 and 12 months – per protocol population

Patient adjusted questionnaire scores at 6 and 12 months – per protocol population

Questionnaire	Control Mean (SE) N	Intervention Mean (SE) N	Difference (SE)	95% CI of the difference	p-value	Adjusted ICC Control	Adjusted ICC Intervention
**6 months:**							
GHQ-12	14.0 (0.69) 228	14.8 (0.78) 248	-0.8 (0.76)	(-2.3, 0.7)	0.280	0	0.058
Barthel Index	15.7 (0.35) 228	15.4 (0.34) 244	0.3 (0.33)	(-0.3,1.0)	0.310	0	0
EQ-5D	0.57 (0.028) 223	0.56 (0.025) 228	0.01 (0.026)	(-0.04, 0.07)	0.602	0.007	0
FAI	18.9 (0.90) 225	17.9 (0.87) 467	1.0 (0.87)	(-0.7, 2.7)	0.240	0	0.011
**12 months:**							
GHQ-12	13.8 (0.70) 201	12.9 (0.85) 227	0.8 (0.85)	(-0.9, 2.5)	0.336	0	0.097
Barthel Index	15.6 (0.35) 203	15.6 (0.36) 226	0.0 (0.37)	(-0.8, 0.7)	0.933	0	0.036
EQ-5D	0.55 (0.033) 199	0.52 (0.032) 215	0.03 (0.034)	(-0.03, 0.1)	0.328	0.034	0.046
FAI	20.0 (0.93) 201	18.2 (0.95) 225	1.7 (0.94)	(-0.1, 3.6)	0.069	0	0.016

Table IX Carer adjusted questionnaire scores at 6 and 12 months – per protocol population

Carer adjusted questionnaire scores at 6 and 12 months – per protocol population

Questionnaire	Control Mean (SE) N	Intervention Mean (SE) N	Difference (SE)	95% CI of the difference	p-value	Adjusted ICC Control	Adjusted ICC Intervention
**6 months:**							
GHQ-12	12.6 (0.82) 58	14.0 (0.90) 67	-1.4 (1.04)	(-3.5, 0.7)	0.181	0	0
CBS	45.5 (2.64) 57	49.7 (2.25) 67	-4.3 (3.02)	(-10.2, 1.7)	0.162	0.073	0.037
**12 months:**							
GHQ-12	13.7 (1.18) 51	14.2 (0.95) 61	-0.50 (1.34)	(-3.2, 2.1)	0.707	0.145	0
CBS	45.1 (2.80) 51	50.1 (2.46) 60	-5.0 (3.22)	(-11.4, 1.3)	0.120	0.054	0.025

Figure II: Adjusted control service differences of GHQ-12 scores in relation to adjusted control score by service completion of time logs

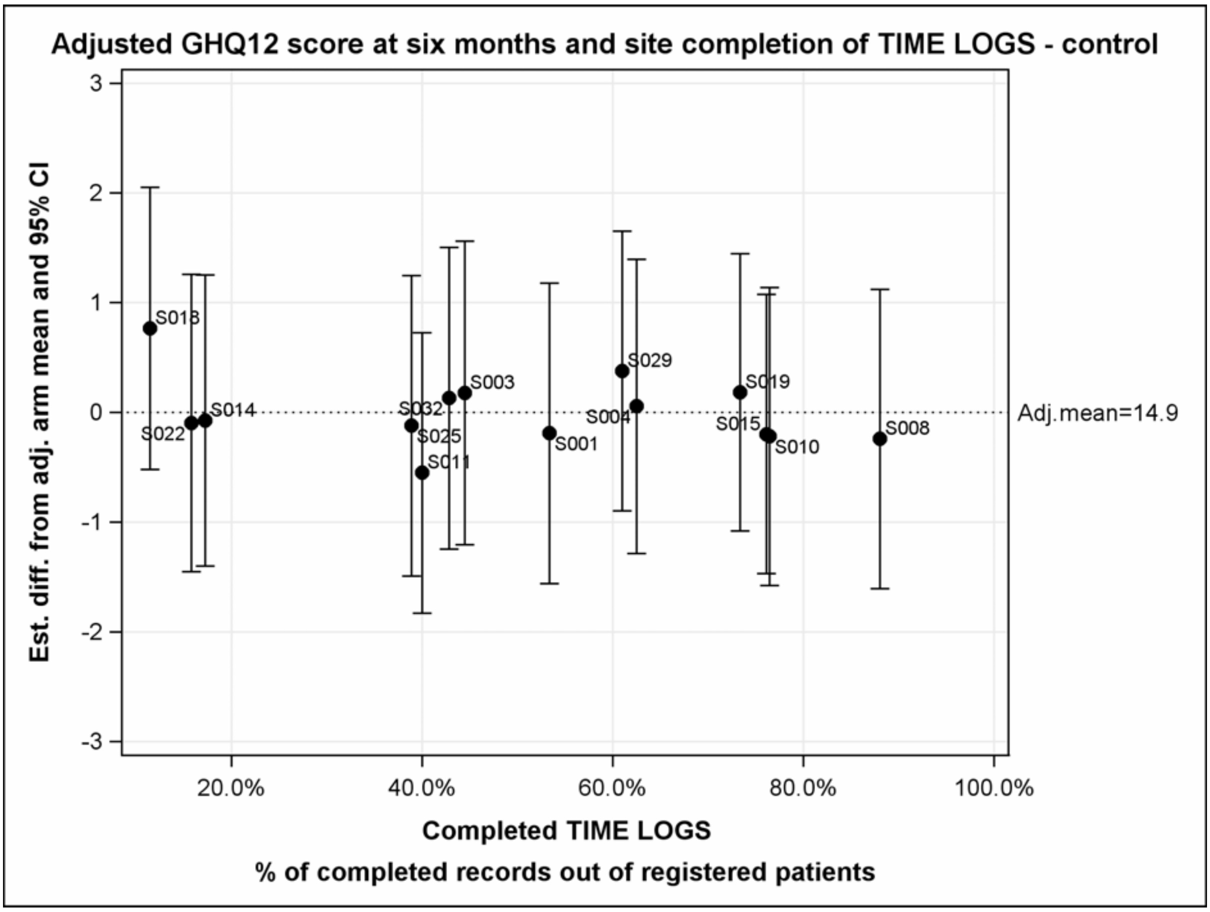

No linear trend was observed between a service's adjusted mean primary outcome and mean percentage of completed time logs.

Figure III Adjusted intervention service differences of GHQ-12 scores in relation to adjusted intervention score by service compliance with intervention

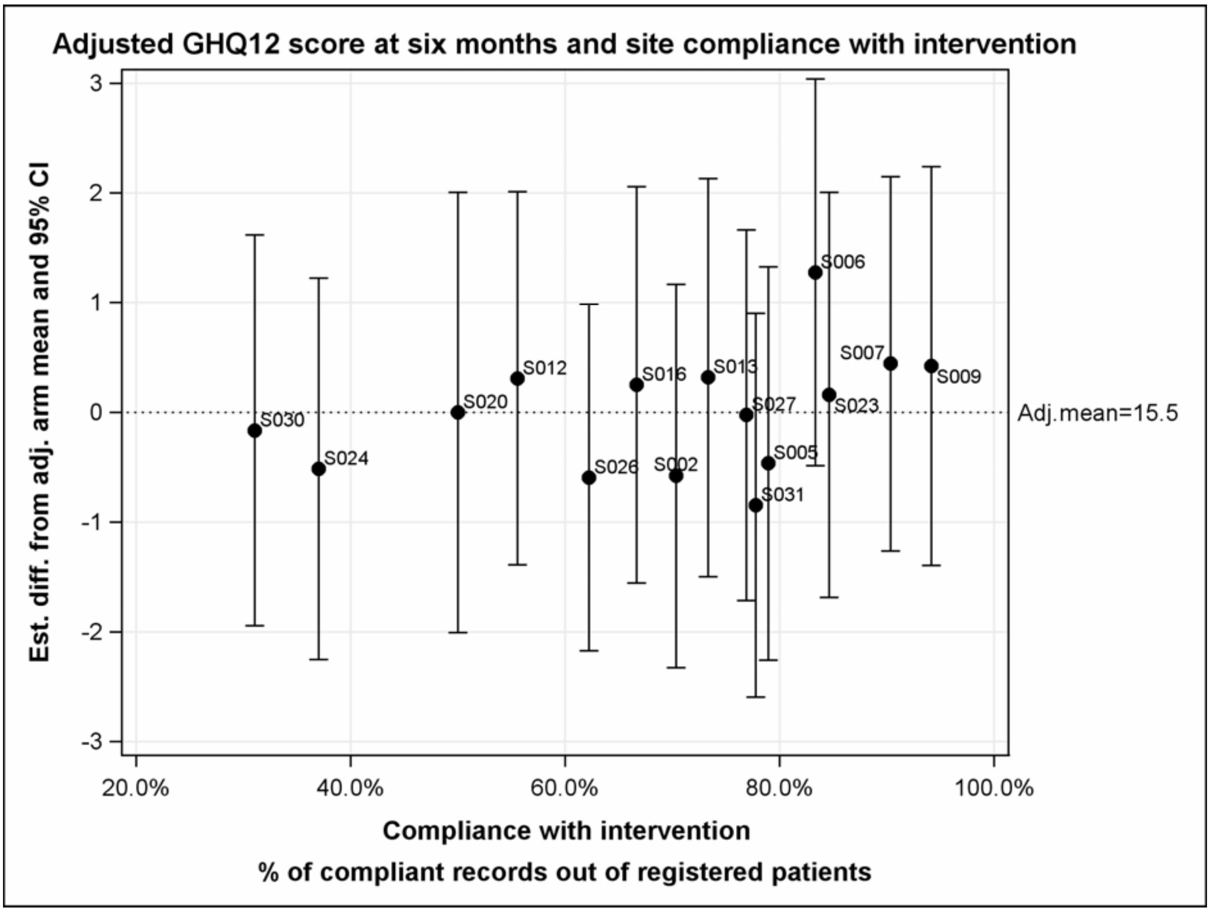

No linear trend was observed between a service's adjusted mean primary outcome and mean percentage of compliant care plans

Economic evaluation methods

Perspective

The economic evaluation was undertaken from two perspectives: (a) health and social care and (b) societal. Health and social care costs included: nursing/residential care; hospital inpatient, outpatient, day hospital and accident and emergency services; primary care; community based health services; and social care services. Societal costs included all of these categories plus informal care costs.

Time horizon

In keeping with the outcome evaluation, the primary endpoint was 6 months. We further examined costs and outcomes at 12 months and over one year (one year costs and QALYs were the sum of values at 6 and 12 months).

Resource use data

Resource use data were collected by self-report at the individual-level using appropriately tailored versions of schedules used in previous stroke studies.[1,2] These were administered alongside other measures at baseline (with reference to the previous three months), 6 and 12 months (with reference to the previous 6 months).

We also prospectively measured the duration of SCC inputs (of both a contact and non-contact nature) at the individual patient level in both intervention and control groups. In the intervention group, these inputs were measured as part of the Care Plan. In the control group, staff recorded equivalent inputs on a specifically designed Time Log. Pay bands were also recorded to enable cost estimation by staff level. Data from the SCC surveys were used for service-relevant imputation values in the event of missing or partially completed Care Plans or Time Logs.

Costs

Individual-level resource use quantities were combined with unit costs (summarised in Table X; further details available upon request) at 2010/2011 prices to calculate a cost per participant. Costs are shown in English pounds sterling (£), and can be converted to United States dollars using the rate £1 = $1.43, based on 2011 purchasing power parities which equalise the purchasing power of the currencies.[3] Discounting was not necessary since the evaluation period did not exceed one year.

Table X: Unit cost summary

Category	Unit	Unit cost (£, 2010/11)
Residential care home stay	Night	75
Nursing home stay	Night	76
Inpatient services	Bed day	Range 315 to 1213
Day hospital/day case services	Activity	Range 230 to 1190
Outpatient services	Visit	Range 3 to 772
Primary care/community-based services	Contact/hour/item	Range 9 to 152
Value of carer time – average wage	Hour	15
Value of carer time – leisure time	Hour	5
Stroke care co-ordinator	Hour	Range 19 to 78
Stroke multi-disciplinary meeting	Hour	284

Quality-adjusted life years

We estimated QALYs from EQ-5D[4] health state measurements at baseline, 6 months and 12 months. We attached utility weights from a United Kingdom general population survey[5] to health states at each time point, with appropriate adjustments for the period of time involved, and estimated QALY gains using linear interpolation to calculate the area under the QALY curve.

Cost–effectiveness and cost–utility analyses

Given two cost perspectives and two outcomes, we examined the following four cost-outcome combinations at the primary endpoint of 6 months: (a) GHQ-12 and health and social care costs (b) GHQ-12 and societal costs (c) QALYs and health and social care costs and (d) QALYs and societal costs. Further examining costs and outcomes at 12 months and over one year led to total of 12 cost-outcome combinations to consider. We only planned to estimate incremental cost-effectiveness ratios (ICERs) for cost-outcome combinations where one group had both higher costs and better outcomes. Cost-effectiveness planes and cost-effectiveness acceptability curves (CEACs) (see below) were only examined for the primary endpoint.

We plotted between-group mean differences in total health and care costs, QALY gains and GHQ-12 scores at 6 months on cost-effectiveness planes. Differences were calculated using bootstrapped regressions (5000 replications; Stata version 10.1) with an adjustment for cluster and the same patient level baseline covariates as used for other group comparisons: Barthel Index (pre- and post- stroke), gender, age, living circumstances (living alone vs. with carer), stroke severity as represented by speech and language impairment (normal/ impaired) and 6CIT score (normal/ impaired), utility score and GHQ-12 score (and total cost from the relevant perspective for the comparison of costs).

CEACs are an alternative to confidence intervals around ICERs. We constructed these to show the probability of the system of care being cost-effective compared to usual care for a range of values that a decision maker may be willing to pay for an additional unit gain in the GHQ-12 or QALYs. CEACs were based on the net benefit approach. Net benefits provide a single summary monetary measure of costs and outcomes for each individual (removing the need to examine ICERs which carry limitations of being based on point estimates and difficulties in estimating confidence intervals around them). Net benefits account for the value (λ) that a decision maker would be willing to pay for a greater net benefit, and are calculated as follows: net benefit = (λ x outcome) – cost. For each cost-outcome combination, we calculated a series of net benefits for a range of relevant λ values (£0 to £2000 per point improvement on the GHQ-12 and £0 to £50,000 per QALY gain). Net benefits were then compared by randomisation group using bootstrapped regressions (5000 replications) of study group upon net benefit, with an adjustment for cluster and the same patient level baseline covariates as used for other group comparisons: Barthel Index (pre- and post- stroke), gender, age, living circumstances (living alone vs. with carer), stroke severity as represented by speech and language impairment (normal/ impaired) and 6CIT score (normal/ impaired), utility score, GHQ-12 score and total cost from the relevant perspective. For each value of λ, the proportion of iterations indicating a higher net benefit for the intervention group were calculated and plotted as a CEAC.

Statistical analyses

All cost and QALY data are reported as mean values with standard deviations. To accommodate a cluster randomisation design, differences in costs and QALYs between groups were tested by multi level modelling using the xtreg procedure in Stata 10.1, from which we report 95% confidence intervals and p values for the differences in means. Relevant baseline patient characteristics were included as covariates for comparisons at follow-ups: Barthel Index (pre- and post- stroke), gender, age, living circumstances (living alone vs. with carer), stroke severity as represented by speech and language impairment (normal/ impaired) and 6CIT score (normal/ impaired), utility and GHQ-12 score (and total cost from the relevant cost perspective for the comparison of costs). Individuals were analysed according to the group to which they were randomised regardless of compliance with the intervention. Resource use differences were not compared statistically to avoid problems associated multiple testing.

Missing data

The base case evaluation was a completers' analysis without imputation for loss of follow-up under the assumption that loss of follow-up was at random. Missing GHQ-12 and EQ-5D data were not imputed. There were inevitably some missing items on returned resource use questionnaires and to allow computation of total costs that reflected variations in resource use rather than variations in data completeness, we imputed missing cost items for returned questionnaires. We imputed a zero cost where there was no indication of a particular resource being used. Where there was indication of use but quantity was missing, we imputed using the mean cost for participants with data for that item at the same assessment point and in the same randomisation group (or the other randomisation group if there were no valid cases to impute from in the same group); in the case of hospital admissions, we used an average admission cost from NHS reference costs, rather than values from within the dataset. All such imputations were made to cost estimates, rather than to the resource use data, so descriptions of resource use data include no imputations.

It was similarly necessary to impute missing data related to SCC inputs; we did this for each SCC component separately to utilise, rather than over-ride, any available data. We assumed a zero cost if a care plan/time log was not completed for the following reasons: referral not received by SCC service, patient declined SCC service, patient died prior to SCC service, SCC unable to contact patient or patient withdrew from all follow-up. For partially completed care plans/time logs, missing duration of inputs were imputed using values from the SCC survey where available (by service) or else the within-group mean cost for those with care plans/time logs and data for the relevant component. Where SCC services were received but a care plan/time log was not completed, we imputed the mean cost for those with data in the control group (the care plan was part of the intervention so providing SCC inputs without it was assumed equivalent to control services). For consenting carers in the intervention group, we assumed a zero cost if an assessment did not take place, if it was unknown whether one took place or if there was no consenting carer. If an assessment took place but duration was missing, we allocated the mean cost for those with completed assessments and data for that component. Data for inputs to non-consenting carers were found to be complete across all components. We assumed a zero cost in the absence of any identified non-consenting carer.

Sensitivity analyses

We examined the effect of loss of follow-up by imputing missing health and social care costs and QALYs at the primary endpoint, 6 months, and checking whether conclusions related to

group differences agreed with the base case analyses (using the same covariates for estimation of mean differences). We re-computed alternative ICERs and CEACs only if different cost or outcome conclusions were suggested. For those lost to follow-up due to death, we computed QALY gains assuming a utility value of zero at 6 months and included costs of SCC inputs whilst assuming all other health and social care costs were zero. For those lost to follow-up for any other reason, we imputed total costs and QALYs using the multiple imputation procedure in Stata 10.1. Imputations were based on key baseline variables expected to predict follow-up costs and QALYs. These were the same variables used as covariates for the comparisons of costs and QALYs for those with data, plus randomisation group: at the patient level, baseline Barthel Index (pre- and post- stroke), gender, age, living circumstances (living alone vs. with carer), stroke severity as represented by speech and language impairment (normal/ impaired) and 6CIT score (normal/ impaired), utility, GHQ12 (and total cost from the relevant cost perspective for the prediction of costs); at the stroke unit level, quality of stroke unit (NSA score), referral rate and SCCs working alone vs. within a community MDT.

Economic evaluation results

Completion rates for resource use questionnaire and EQ-5D

Completion rates for the resource use questionnaire and EQ-5D were similar at each assessment point and balanced between the two groups. Although differences were not explored statistically, baseline characteristics of patients with the necessary data at 6 months appeared similar to those of the full sample. Therefore, the completers' analysis is likely to be representative of the full sample.

Resource use

Resource use was broadly comparable between the two groups at each assessment (Tables XI to XIII). For brevity, we report all inpatient services and all informal care plus other health and social care resources used by at least 10% of responders in either trial arm at the relevant assessment; full resource use data are available upon request from Anita Patel.

Economic evaluation references

1. Patel A, Knapp M, Evans A, Perez I, Kalra L. Training care givers of stroke patients: economic evaluation. *BMJ* 2004; 328**:** 1102–1104.
2. Patel A, Knapp M, Perez I, Evans A, Kalra L. Alternative strategies for stroke care: cost–effectiveness and cost–utility analyses from a prospective randomized controlled trial. *Stroke* 2004; 35: 196–203.
3. OECD. StatsExtracts 4. PPPs and exchange rates. (Accessed 22 December 2014 at http://stats.oecd.org/Index.aspx?DataSetCode=SNA_TABLE4)
4. Dolan P, Gudex C, Kind P, Williams A. A social tariff for EuroQol: results from a UK population survey: University of York. 1995.
5. The EuroQol Group. EuroQol: a new facility for the measurement of health related quality of life. *Health Policy* 1990; 16: 199–208.

Table XI: Resource use at baseline (for previous 3 months)[c]

	Unit	Intervention = 401 % users	Mean[a]	SD	Control = 398	Mean[a]	SD
**Index stroke admission[b]**	bed days	99	39	44	99	30	35
**Inpatient services**	bed day	7	11	24	10	7	6
**Accident & emergency**	visit	7	11	24	10	7	6
**Outpatient services**	visit	8	2	4	10	1	1
**Community based services**							
General practitioner							
Surgery visit	visit	49	2	1	44	2	2
Home visit	visit	9	2	1	11	2	2
Telephone call	call	10	2	1	12	2	1
Repeat prescription	occurrence	47	2	1	49	2	1
Practice nurse	visit	26	2	1	23	2	2
Chiropodist	contact	10	2	1	12	1	1
Dentist	contact	12	1	1	12	1	1
Optician	contact	12	1	<1	12	1	<1
**Informal care from co- residents**							
Personal care	hour	3	47	43	3	219	320
Providing transport	hour	3	78	205	6	37	56
Preparing meals	hour	4	138	175	6	134	141
Housework/laundry	hour	4	147	186	6	95	93
DIY	hour	2	97	247	3	40	65
Gardening	hour	3	94	207	4	31	44
Shopping	hour	4	81	184	5	41	47
Outings	hour	2	106	231	3	44	63
Socialising	hour	3	415	604	3	202	428
Help managing finances	hour	3	77	214	4	31	40
**Informal care from non-residents**							
Personal care	hour	1	39	29	1	171	281
Providing transport	hour	6	25	23	4	39	48
Preparing meals	hour	3	66	72	2	48	65
Housework/laundry	hour	4	43	39	4	24	20
DIY	hour	3	19	19	2	10	7
Gardening	hour	4	18	14	3	18	14
Shopping	hour	5	24	15	4	28	21
Outings	hour	5	28	56	3	23	16
Socialising	hour	5	95	115	5	78	100
Help managing finances	hour	2	19	14	2	12	7

a. Mean for valid user values only
b. Not included in the evaluation.
c. Inpatient services and informal care plus other resources used by at least 10% of either group.

Table XII: Resource use at 6 months (in previous 6 months)[c]

		Intervention = 307			Control = 295		
	Unit	% users	Mean[a]	SD	% users	Mean[a]	SD
**Inpatient Services**	bed day	19	15	18	19	14	27
**Day hospital / day cases**	activity	12	1	1	8	1	1
**Accident & emergency**	visit	17	2	2	14	2	1
**Outpatient services**	visit	44	3	3	40	3	5
**Physiotherapist, hospital[b]**	visit	12	8	8	17	7	8
**Community based services**							
General practitioner							
Surgery visit	visit	57	3	2	60	3	2
Home visit	visit	24	2	2	22	2	1
Telephone call	call	20	2	2	21	2	3
Repeat prescription	occurrence	52	3	4	51	5	3
Practice nurse	visit	33	3	3	40	3	3
Physiotherapist	home visit	28	8	9	22	8	10
Occupational therapist	visit	26	5	8	24	6	7
Speech and language therapist	home visit	11	4	3	13	4	4
Social worker	home visit	12	2	1	11	2	1
Community / district nurse	contact	23	7	21	23	4	9
Chiropodist	contact	18	2	2	16	2	1
Dentist	contact	21	2	1	14	1	1
Optician	contact	20	1	1	22	1	1
**Informal care from co-residents**							
Personal care	hour	15	385	834	14	242	324
Providing transport	hour	15	108	140	14	120	126
Preparing meals	hour	19	249	234	14	249	200
Housework/laundry	hour	18	244	305	14	204	163
DIY	hour	9	87	224	4	49	51
Gardening	hour	11	80	94	8	60	52
Shopping	hour	16	115	122	15	95	113
Outings	hour	14	116	142	9	93	87
Socialising	hour	14	728	1125	9	489	488
Help managing finances	hour	13	89	123	11	124	225
**Informal care from non-residents**							
Personal care	hour	8	110	96	6	110	102
Providing transport	hour	14	63	82	14	58	58
Preparing meals	hour	8	104	135	6	85	98
Housework/laundry	hour	9	80	82	7	45	43
DIY	hour	6	20	20	4	24	35
Gardening	hour	9	26	36	7	32	37
Shopping	hour	11	48	48	11	44	35
Outings	hour	13	57	98	11	54	62
Socialising	hour	13	156	163	9	146	218
Help managing finances	hour	7	60	69	5	33	36

a. Mean for valid user values only

b. Separate to other outpatient visits. Further visits to these services are also included in the broader outpatient visit numbers.

c. Inpatient services and informal care plus other resources used by at least 10% of either group.

Table XIII: Resource use at 12 months (in previous 6 months)[c]

	Unit	Intervention = 283			Control = 268		
		% users	Mean[a]	SD	% users	Mean[a]	SD
**Inpatient services**	bed day	16	9	15	15	8	9
**Day hospital / day cases**	activity	8	2	1	10	1	<1
**Accident & emergency**	visit	12	2	1	10	2	1
**Outpatient services**	visit	36	3	3	37	3	5
**Physiotherapist, hospital[b]**	visit	14	6	6	12	6	8
**Community based services**							
General practitioner							
Surgery visit	visit	53	3	3	60	3	2
Home visit	visit	15	3	4	13	3	6
Telephone call	call	18	2	1	13	3	3
Repeat prescription	occurrence	49	5	3	54	5	2
Practice nurse	visit	35	3	4	44	3	4
Community / district nurse	contact	15	8	27	14	4	5
Chiropodist	contact	18	2	1	18	2	2
Dentist	contact	27	2	1	22	2	1
Optician	contact	21	1	<1	24	1	1
**Other services**	occurrence	6	4	6	3	2	1
**Informal care from co- residents**							
Personal care	hour	11	564	1695	6	460	1035
Providing transport	hour	12	202	357	10	134	278
Preparing meals	hour	14	317	336	9	213	203
Housework/laundry	hour	14	339	769	11	210	235
DIY	hour	7	157	455	4	67	83
Gardening	hour	9	137	390	8	81	160
Shopping	hour	11	175	349	10	80	141
Outings	hour	11	173	378	7	123	176
Socialising	hour	11	550	776	7	343	488
Help managing finances	hour	9	169	403	8	63	117
**Informal care from non-residents**							
Personal care	hour	5	209	356	3	186	349
Providing transport	hour	12	53	66	11	42	40
Preparing meals	hour	7	106	170	4	87	84
Housework/laundry	hour	9	107	154	7	60	67
DIY	hour	5	46	43	7	34	44
Gardening	hour	6	38	37	6	31	36
Shopping	hour	8	62	72	8	44	39
Outings	hour	11	63	135	9	47	46
Socialising	hour	12	222	356	8	116	140
Help managing finances	hour	5	49	64	4	44	37

a. Mean for valid user values only

b. Separate to other outpatient visits. Further visits to these services are also included in the broader outpatient visit numbers.

c. Inpatient services and informal care plus other resources used by at least 10% of either group.

Figure IV: **Probability that the intervention is cost effective compared with the control at 6 months from health/social care and societal perspectives, for a range of willingness to pay values for an additional QALY gain**

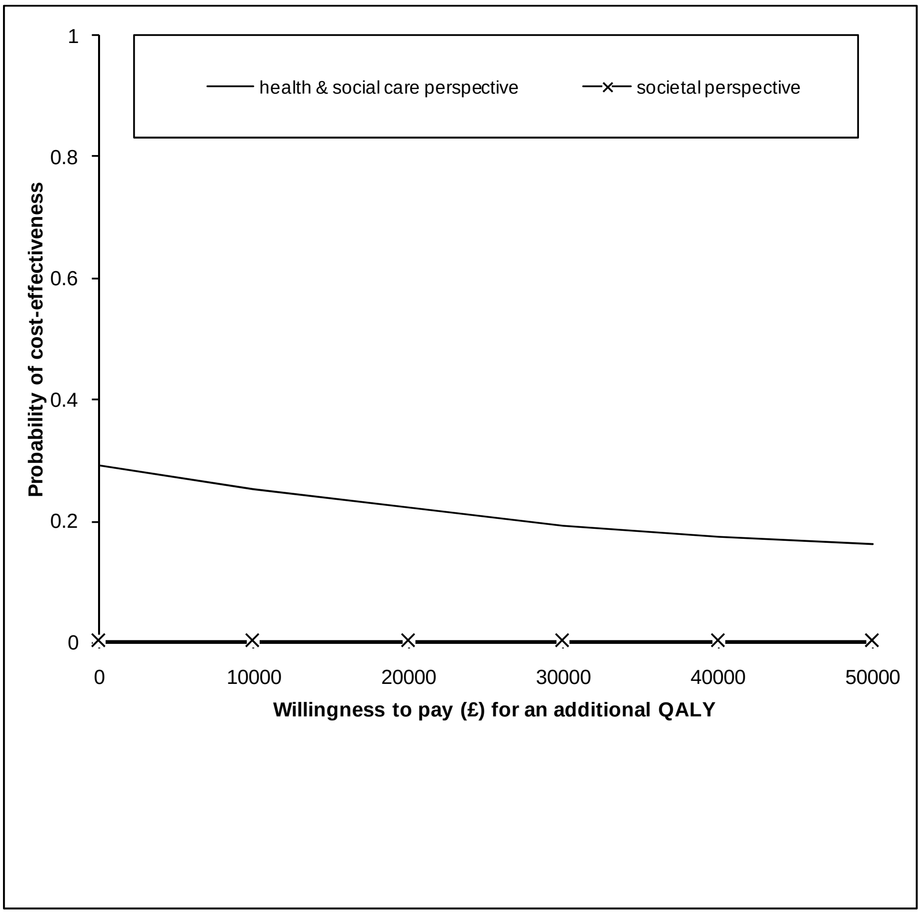

Cost–effectiveness and cost–utility

Figure V: Cost-effectiveness plane of incremental total health and social care costs and point changes on the GHQ-12 at 6 months

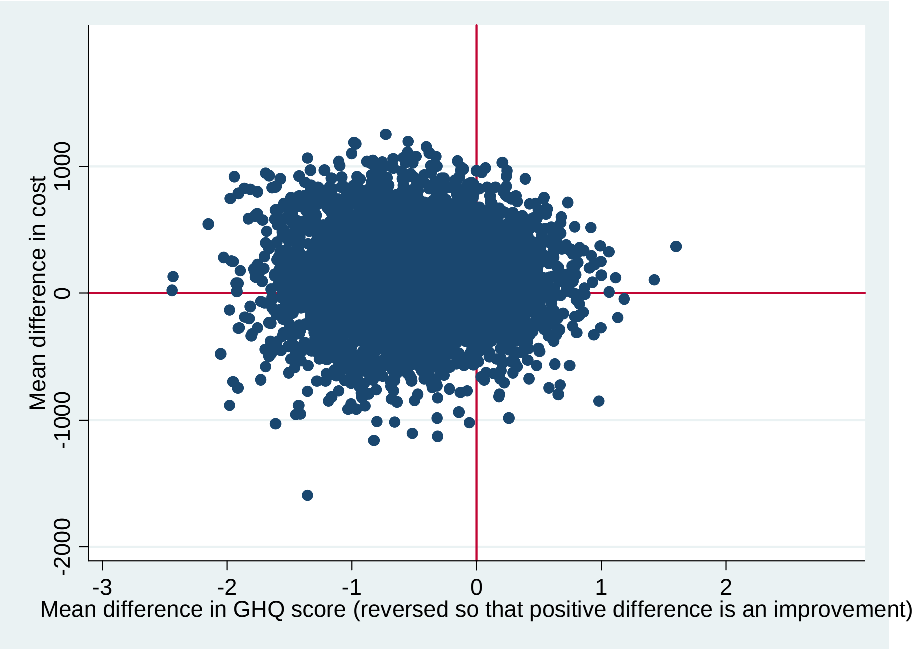

Figure VI: Cost-effectiveness plane of incremental total health and social care costs and QALY gains at 6 months

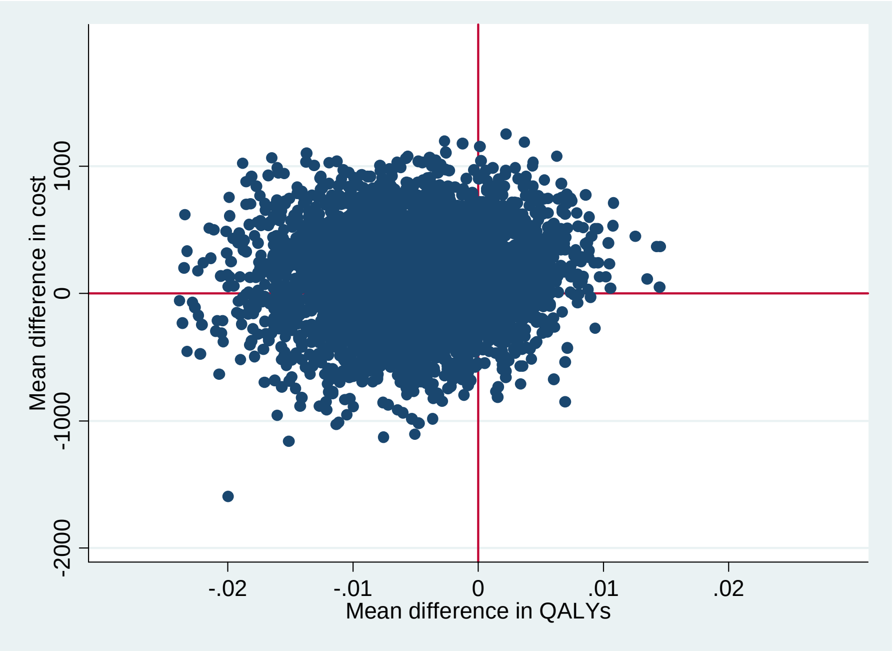

Figure VII: Probability that the intervention is cost effective compared with the control at 6 months, from each cost perspective, for a range of willingness to pay values for an additional point improvement on the GHQ-12

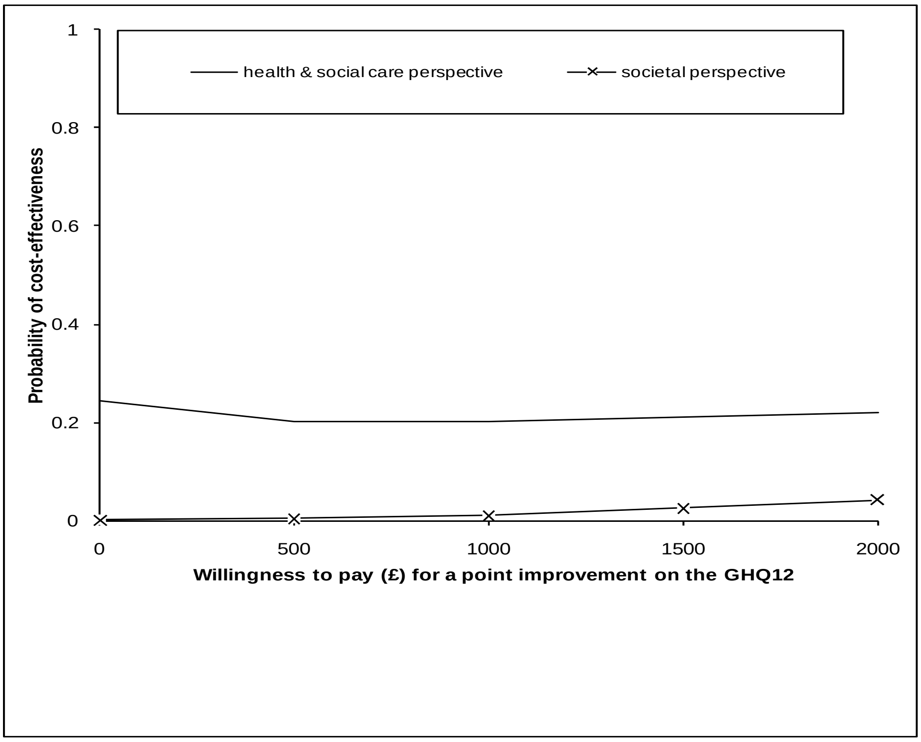

Supplement: Supplementary file 1 [file str-46-2212-s001.pdf]
