# Supplementary material for: Cluster Randomized Controlled Trial: Clinical and Cost-Effectiveness of a System of Longer-Term Stroke Care
Source: Stroke. 2015 Jul 27;46(8):2212–9. doi: 10.1161/STROKEAHA.115.008585 (PMC4512748; doi:10.1161/STROKEAHA.115.008585)
Supplement: Supplementary file 2 [file str-46-2212-s002.pdf]

Advertisement

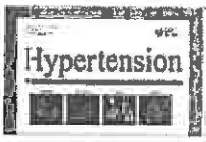

Follow *Hypertension's* "Clinical Implications" Series—
A feature highlighting the clinical relevance of 3 select articles published in this issue.

AHA Journals

Acknowledgment Permission Form

Journal: STROKE

Manuscript No.: STROKE/2014/008585

First author: FORSTER A

Title of work: CLUSTER RANDOMISED CONTROLLED TRIAL: THE CLINICAL AND COST-EFFECTIVENESS OF A SYSTEM OF LONGER-TERM STROKE CARE

Acknowledgment Permission:

Authors must provide written permission/approval from all individuals mentioned by name in the Acknowledgements section of a submitted manuscript. By signing this form, any and all acknowledged persons therefore state that they have read and approved the mention of their names in the Acknowledgment section of the aforementioned paper.

Printed Name: RENEE ROMEO
Signature: [illegible]
Date: 11th March 2015

Printed Name:
Signature:
Date:

Printed Name:
Signature:
Date:

Printed Name:
Signature:
Date:

Abstracts
from AHA Scientific Sessions

Late-Breaking
Basic Science Abstracts
from AHA Scientific Sessions

Advertisement

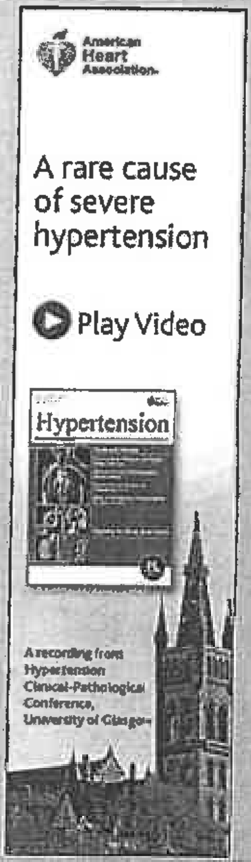

Advertisement
Hypertension
Follow *Hypertension's* "Clinical Implications" Series—
A feature highlighting the clinical relevance of 3 select articles published in this issue.

DONATE HELP CONTACT AHA HOME

User Name
Password
LOGIN

AHA Journals

American Heart Association.

Home • Subscriptions • Feedback • Help • Circulation Journals Home • AHA Journals Home

Search: Go Advanced Search

Acknowledgment Permission Form

Journal: Stroke

Manuscript No.: STROKE/2014/008585

First author: Anne Forster

Title of work: [illegible] randomised controlled trial the clinic

Acknowledgment Permission:

Authors must provide written permission/approval from all individuals mentioned by name in the Acknowledgements section of a submitted manuscript. By signing this form, any and all acknowledged persons therefore state that they have read and approved the mention of their names in the Acknowledgment section of the aforementioned paper.

Printed Name: Margaret Heslin
Signature: [illegible]
Date: 11/3/15

Printed Name:
Signature:
Date:

Printed Name:
Signature:
Date:

Printed Name:
Signature:
Date:

Abstracts
from AHA Scientific Sessions

Late-Breaking
Basic Science Abstracts
from AHA Scientific Sessions

Advertisement
American Heart Association.
A rare cause of severe hypertension
Play Video
Hypertension
A recording from Hypertension Clinical-Pathological Conference, University of Glasgow

Advertisement

Hypertension

Follow *Hypertension's* "Clinical Implications" Series—

A feature highlighting the clinical relevance of 3 select articles published in this issue.

Acknowledgment Permission Form

Journal: STROKE

Manuscript No: STROKE/2014/008585

First author: Anne Forster PhD

Title of work: LoTS Care Stroke System of Care Trial

Acknowledgment Permission

Authors must provide written permission/approval from all individuals mentioned by name in the Acknowledgements section of a submitted manuscript. By signing this form, any and all acknowledged persons therefore state that they have read and approved the mention of their names in the Acknowledgment section of the aforementioned paper.

Printed Name: Alison Fergusson

Signature: [illegible]

Date: 11 MAR 2015

Printed Name: Andrew Carter

Signature: [illegible]

Date: 11/03/2015

Printed Name: Nicholas Hearfield

Signature: [illegible]

Date: 11/03/2015

Printed Name: [illegible]

Signature:

Date:

Abstracts from AHA Scientific Sessions

Late-Breaking Basic Science Abstracts from AHA Scientific Sessions

Advertisement

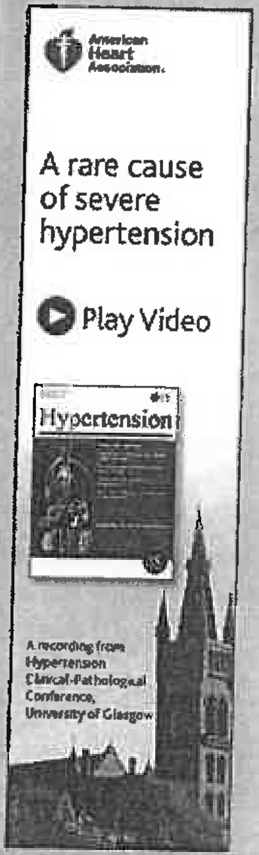

Acknowledgment Permission Form

Journal Stroke
Manuscript No. 2014/008585
First author Forster A
Title of work Cluster randomised controlled trial: the clinical and cost-effectiveness of a system of longer-term stroke care.

Acknowledgment Permission:

Authors must provide written permission/approval from all individuals mentioned by name in the Acknowledgements section of a submitted manuscript. By signing this form, any and all acknowledged persons therefore state that they have read and approved the mention of their names in the Acknowledgment section of the aforementioned paper.

Printed Name Professor Tony Rudd
Signature [illegible]
Date 3/12/14
Printed Name
Signature

Acknowledgment Permission Form

Journal Stroke
Manuscript No. 2014/008585
First author Forster Anne
Title of work Cluster randomised controlled trial the clinical and cost-effectiveness of a system of longer-term stroke care

Acknowledgment Permission:

Authors must provide written permission/approval from all individuals mentioned by name in the Acknowledgements section of a submitted manuscript. By signing this form, any and all acknowledged persons therefore state that they have read and approved the mention of their names in the Acknowledgment section of the aforementioned paper.

Printed Name Allan House
Signature [illegible]
Date 1 Dec 2014
Printed Name
Signature
Date
Printed Name
Signature

American Heart Association
Password LOG IN

AHA Journals

Acknowledgment Permission Form

Journal stroke
Manuscript No. 2014/008585
First author Forster
Title of work Cluster randomised controlled trial the clinical and cost-effectiveness of a system of longer-term stroke care

Acknowledgment Permission:

Authors must provide written permission/approval from all individuals mentioned by name in the Acknowledgements section of a submitted manuscript. By signing this form, any and all acknowledged persons therefore state that they have read and approved the mention of their names in the Acknowledgment section of the aforementioned paper.

Printed Name Peter Wanklyn
Signature [illegible]
Date 9/12/14
Printed Name
Signature
Date
Printed Name
Signature

Abstracts from AHA Scientific Sessions
Basic Science Abstracts from AHA Scientific Sessions
American Heart Association
A rare cause of severe hypertension
Play Video
